# Holistic Approach of Swiss Fetal Progenitor Cell Banking: Optimizing Safe and Sustainable Substrates for Regenerative Medicine and Biotechnology

**DOI:** 10.3389/fbioe.2020.557758

**Published:** 2020-10-23

**Authors:** Alexis Laurent, Nathalie Hirt-Burri, Corinne Scaletta, Murielle Michetti, Anthony S. de Buys Roessingh, Wassim Raffoul, Lee Ann Applegate

**Affiliations:** ^1^Regenerative Therapy Unit, Lausanne University Hospital, University of Lausanne, Épalinges, Switzerland; ^2^Tec-Pharma SA, Bercher, Switzerland; ^3^LAM Biotechnologies SA, Épalinges, Switzerland; ^4^Children and Adolescent Surgery Service, Lausanne University Hospital, University of Lausanne, Lausanne, Switzerland; ^5^Plastic, Reconstructive and Hand Surgery Service, Lausanne University Hospital, University of Lausanne, Lausanne, Switzerland; ^6^Oxford Suzhou Center for Advanced Research, Science and Technology Co., Ltd., Oxford University, Suzhou, China; ^7^Competence Center for Applied Biotechnology and Molecular Medicine, University of Zurich, Zurich, Switzerland

**Keywords:** biotechnology, cell therapy, clinical cell banking, fetal cell transplantation, primary fetal progenitor cells, regenerative medicine

## Abstract

Safety, quality, and regulatory-driven iterative optimization of therapeutic cell source selection has constituted the core developmental bedrock for primary fetal progenitor cell (FPC) therapy in Switzerland throughout three decades. Customized Fetal Transplantation Programs were pragmatically devised as straightforward workflows for tissue procurement, traceability maximization, safety, consistency, and robustness of cultured progeny cellular materials. Whole-cell bioprocessing standardization has provided plethoric insights into the adequate conjugation of modern biotechnological advances with current restraining legislative, ethical, and regulatory frameworks. Pioneer translational advances in cutaneous and musculoskeletal regenerative medicine continuously demonstrate the therapeutic potential of FPCs. Extensive technical and clinical hindsight was gathered by managing pediatric burns and geriatric ulcers in Switzerland. Concomitant industrial transposition of dermal FPC banking, following good manufacturing practices, demonstrated the extensive potential of their therapeutic value. Furthermore, *in extenso*, exponential revalorization of Swiss FPC technology may be achieved *via* the renewal of integrative model frameworks. Consideration of both longitudinal and transversal aspects of simultaneous fetal tissue differential processing allows for a better understanding of the *quasi*-infinite expansion potential within multi-tiered primary FPC banking. Multiple fetal tissues (e.g., skin, cartilage, tendon, muscle, bone, lung) may be simultaneously harvested and processed for adherent cell cultures, establishing a unique model for sustainable therapeutic cellular material supply chains. Here, we integrated fundamental, preclinical, clinical, and industrial developments embodying the scientific advances supported by Swiss FPC banking and we focused on advances made to date for FPCs that may be derived from a single organ donation. A renewed model of single organ donation bioprocessing is proposed, achieving sustained standards and potential production of billions of affordable and efficient therapeutic doses. Thereby, the aim is to validate the core therapeutic value proposition, to increase awareness and use of standardized protocols for translational regenerative medicine, potentially impacting millions of patients suffering from cutaneous and musculoskeletal diseases. Alternative applications of FPC banking include biopharmaceutical therapeutic product manufacturing, thereby indirectly and synergistically enhancing the power of modern therapeutic armamentariums. It is hypothesized that a single qualifying fetal organ donation is sufficient to sustain decades of scientific, medical, and industrial developments, as technological optimization and standardization enable high efficiency.

## Introduction

### Evolution of Regenerative Medicine and Cell Therapies

Changes in demographics and lifestyle worldwide lead to drastic modifications in the incidence and prevalence of degenerative diseases afflicting the musculoskeletal system and cutaneous structures in general. Accidental trauma continuously yields high numbers of acute clinical cases necessitating considerable therapeutic resources. High demand for efficacious preventive and curative treatments has prompted increasing effort and resource allocation in translational medical research and development. A specific focus area has been the development and implementation of innovative products or protocols designed to optimize biological functions or complement traditional surgical management (Déglise et al., 1987; Vacanti and Langer, 1999; Marks and Gottlieb, 2018). In this context, regenerative medicine currently diversifies into vast arrays of novel therapeutic leads, among which cell therapies and cell-based products represent prime prospects. Indeed, such therapies or products, initially proposed over a century ago by Dr. Brown-Séquard and later by Dr. Niehans in Switzerland, constitute multiple potential clinical implementations toward tissue repair optimization and normal organ and system function restoration (Abdel-Sayed et al., 2019b). The reconstitution of maximal patient health can be efficiently implemented through synergistic combinations of tissue engineering, specialized surgical techniques, or classical pharmaco-therapeutic management (Montjovent et al., 2004; Bach et al., 2006; Loebel and Burdick, 2018; Costa-Almeida et al., 2019).

### Importance of Standardized Therapeutic Cell Sources

For classic and novel biological active pharmaceutical ingredients (API), preliminary considerations and prerequisites for biological product development or cell therapy studies reside in the starting materials and cell sourcing. Numerous heterogeneous biological sources have been considered by researchers in human regenerative medicine. Autologous, allogenic, or xenogenic biopsies of various developmental stages may be processed, whereas specific cultured progeny cells retain inherent multifactorial problems to be projected in therapeutic product development processes. Imperative technical, biological, clinical, and sustainability considerations, therefore, help condition and orient cell source selection procedures. Requirements for potential transformation and widespread therapeutic use comprise safety and consistency, availability in adequate quantities, traceable characterization, sufficient inherent expansion capacity, and compatibility with acceptable delivery methods such as engineered bioscaffolds (Doyle and Griffiths, 1998; Monti et al., 2012). Diverse classes of cell sources fit these restrictive criteria, including, but without being limited to, fetal progenitor cells (FPC), embryonic stem cells (ESC), adult stem cells [adipose stem cells (ASC), bone marrow-derived mesenchymal stem cells (BM-MSC), itMSC (ischemia-tolerant mesenchymal stem cells)], neural stem cells (NSC), limbal stem cells (LSC), hematopoietic stem cells (HSC), endothelial progenitor cells (EPC), umbilical cord cells, neonatal foreskin cells, platelets, placenta, and amniotic fluid cells (Vertelov et al., 2013; Heathman et al., 2015; Mount et al., 2015; Muraca et al., 2017; Li and Maitz, 2018; Sacchetti et al., 2018; Jayaraj et al., 2019; Torres-Torrillas et al., 2019). Most available cell sources are technically demanding, as progeny cells require dedicated processing or biochemical manipulation to orient or stabilize their potency and self-renewal capacity. Technical limitations related to sub-optimal intrinsic biological parameters significantly hinder the development of therapeutic cellular products. Increased complexity and costs have belated the development or lengthened the pathways for product market approvals (Heathman et al., 2015; Mount et al., 2015). Potential obstacles comprise low cell proliferation potential, the relative scarcity of the source within donors, high phenotypic plasticity or highly variable differentiation potential, tendency to serve as a communicable disease vector, or mediocre *in vitro* stability and lifespan (Rayment and Williams, 2010; Ratcliffe et al., 2011; Abbasalizadeh and Baharvand, 2013; Heathman et al., 2015; Hunsberger et al., 2015).

### Allogenic FPC Technology for Translational Research

Pragmatic optimization of cell source selection and processing is crucial within translational development and clinical implementation of cell therapies and related products. Iterative amelioration and successful application of standardized workflows have led to identify allogenic primary FPC sources as highly promising and efficient candidates for regenerative medicine (Hebda and Dohar, 1999; De Buys Roessingh et al., 2006; Mirmalek-Sani et al., 2006; Metcalfe and Ferguson, 2007, 2008; Larijani et al., 2015; Grognuz et al., 2016b; Kim et al., 2018). Upon adequate isolation from fetal tissues (i.e., enzymatic or mechanical methods), culture-expansion and cryopreservation, progeny cells and derivatives present numerous advantages. Fetal progenitor cells differentiate until acquiring stable phenotypic (i.e., tissue-specific) characteristics, while retaining intrinsic feeble immunogenic potential, high longitudinal expansion capabilities, and potent stimulatory effects (Quintin et al., 2007; Laurent et al., 2020d). Additionally, such cell types possess few growth requirements to establish an adherent monolayer culture, have high cytocompatibility with various bio-constructs, are resistant to oxidative stress, and have trophic or paracrine mediator effects toward scarless wound healing (Shah et al., 1994; Cass et al., 1997; Doyle and Griffiths, 1998). Furthermore, validation of consistent and robust FPC banking at an efficient industrial scale following good manufacturing practices (GMP) is enabled by continued evaluation of sterility, safety, identity, purity, potency, stability, and efficacy (Quintin et al., 2007). Such prerequisite characteristics defined under restrictive regulations and quality standards for biologicals and starting materials for cell therapies or cell-based products must be investigated rapidly within product development pathways (Doyle and Griffiths, 1998). Allogenic FPC therapies may therefore demonstrably minimize delays in medicinal product availability, as extensive cell banks may serve for direct clinical application or further product developments. Although certain FPCs have yet to demonstrate potential performance advantages when compared to adult cell types in large *in vivo* settings, clinical insights from the past two decades in our Lausanne Burn Center have outlined the superiority of dermal FPCs versus standard cell therapy products and therapies in use (i.e., autologous platelet-rich plasma, cultured epithelial autografts, cultured dermal-epidermal autografts). Multiple clinical trials in Switzerland and in Asia (i.e., Japan, Taiwan) have confirmed the potential for diversified therapeutic uses of dermal FPCs (e.g., FE002-SK2 cell type) as cell therapies. Additionally, our group has three decades of clinical experience with cell-based cell-free topical formulations (i.e., ovine FPC-based cell-free products) classified as cosmetics or medical devices, which were and are used by clients and patients around the world, with positive feedback related to numerous diversified cutaneous affections.

### Translation, Industrial Development, and Commercialization of Swiss FPC Technology

Cell therapies have been the focus of many public and private sponsors, whereas successful development is highly dependent on interprofessional collaboration integrating all complementary dimensions of novel products and protocols (Marks and Gottlieb, 2018). Allogenic cell-based therapies comprising cell culture steps may be classified as advanced therapy medicinal products (ATMP), and derivatives, as medical devices, whereas using correctly harnessed, consistent, and robust cell sources yields enormous advantages (Applegate et al., 2009; Marks and Gottlieb, 2018). Indeed, fundamental safety and traceability elements are required to prepare investigational medicinal product dossiers (IMPD) and investigator’s brochures (IB), whereas optimal biological starting materials may be procured and processed through well-defined Fetal Transplantation Program workflows (Rayment and Williams, 2010; Heathman et al., 2015; Laurent et al., 2020f). Additionally, the robustness of multi-tiered primary FPC biobanks ensures optimal and cost-effective manufacturing for processes which require biological material sourcing. Pragmatic devising and implementation of Fetal Transplantation Programs can realistically be achieved in less than six months, with investment costs around a million Swiss Francs (CHF), to establish a GMP parental cell bank (PCB). Assuming total valorization of progeny cellular materials, industrial development efforts may be sustainably equipped for decades and potentially generate trillions of CHF in revenues following a single organ donation. In addition, direct costs of active principles (i.e., viable cells or cell-free extracts) are negligible within market-approval and commercialization steps of standardized bioengineered therapeutic agents. Unique conjunctures of high innovation and local incentives toward industrial development and commercialization of life science products in Western Switzerland (i.e., Health Valley) have led to the development and marketing of Swiss FPC banking and therapeutic/regenerative derivatives in the past decades. Swiss FPC technology is well adapted to tackle regulatory and industrial manufacturing challenges, while safely and effectively supplying arrays of core and adjuvant therapeutic components for highly innovative Swiss-made products globally. Notably, several patents and two University Hospital spin-offs (i.e., ELANIX Sàrl and Neocutis SA) have contributed to translational developments or commercialization of tissue engineering products (TEPs) or cosmeceutical products around the world.

### Hypothesis Formulation: One-Shot Fetal Transplantation Program

Optimal management of safety and consistency of therapeutic cell sources is attained by avoiding the pooling of numerous heterogeneous biological samples. Therefore, pragmatic devising and exploitation of Fetal Transplantation Programs present unique characteristics and considerable advantages, outlined throughout two decades of translational research on FPCs in Switzerland. Indeed, ethical and controlled revalorization of a single qualifying therapeutically aborted fetus and donated tissues enables, in a unique way, the differential and simultaneous establishment of multiple primary FPC types (e.g., derived from skin, cartilage, tendon, muscle, lung, bone, connective tissue, intervertebral disc). Furthermore, such transversal conceptual approaches to biobanking have been successfully experimentally validated and iteratively optimized for human, equine, and ovine FPC types in Switzerland (Table 1; Applegate et al., 2013; Laurent et al., 2020b,e). Thereby, each individual and tissue-specific cell source may be selectively applied to complementary cutaneous or musculoskeletal regenerative medicine applications and biotechnological developments. Here, we integrated fundamental, preclinical, clinical, and industrial implementational developments representing the scientific advances supported by multi-tiered FPC banking in Switzerland. Overall, cultured FPCs appear as optimal fits for modern regulatory framework development and stringent GMP industrial transposition in a rapid, safe, effective, and traceable manner (Laurent et al., 2020e,g). The benefit of the Swiss FPC technology described herein is the safe, standardized, ethical, and continual high-value supply chain design for unique diversified biological assets. It is hypothesized that a single qualifying fetal organ donation is sufficient to sustain decades of scientific, medical, and industrial developments, as related technological optimization and standardization enable high efficiency. The range of possible valorization applications levels with the *quasi*-indefinite potential material yield of multi-tiered FPC biobanks. The core therapeutic value of optimized and comprehensive Fetal Transplantation Programs enables sustainable and widespread treatment of millions of patients suffering from cutaneous and musculoskeletal diseases with affordable and effective therapeutic products. The main goal of this work was to substantiate, convey, and broaden awareness and interest around the use of standardized protocols for translational regenerative medicine utilizing FPCs. The renewed transversal and longitudinal model of single organ donation bioprocessing described herein shall continue to provide persistent contributions to modern translational regenerative medicine and biopharmaceutical therapeutic product manufacturing, increasing the power of modern therapeutic armamentariums. An overview of implemented therapies used for managing burns and wounds over the past two decades will be highlighted. In addition, progress on characterization and preclinical work on other tissue-specific FPC types will be reviewed, in order to show parallels in pathways to implement new clinical treatments.

**TABLE 1:** Overview of primary FPC types established and studied within the Swiss FPC Transplantation Programs, with respective applications and gathered experiences.

FPC types	Scope of work and gathered experience	Cell type lifespan characteristics	Selected references
Human dermal FPCs (e.g., FE002-SK2 cell type)	The most clinical experience around cutaneous tissue regeneration has been gathered using such cell types, effectively applied for managing severe burns, refractory ulcers, or donor-site wounds. Safety and efficacy of such therapeutic materials have been demonstrated in various clinical trials. Thorough experience has been gathered around industrial GMP manufacturing transposition for commercialization of cell-based or cell-derivative products. The extensive industrial biobanking potential was validated using the FE002-SK2 cell type	In preclinical works, FE002-SK2 cells were studied up to P18–P20 In clinical settings, FE002-SK2 EOPCBs were established and validated at P12 Current clinical protocols describe the use of cells at P8 When using the same isolation and culture methods as described for FPCs, adult dermal fibroblasts are generally characterized by a lifespan of 6–7 passages	Hohlfeld et al., 2005 Quintin et al., 2007 Hirt-Burri et al., 2011 De Buys Roessingh et al., 2015 Laurent et al., 2020e,h
Human tendon FPCs (e.g., FE002-Ten cell type)	Such cell types have been extensively characterized *in vitro* and were shown to optimally adapt to drug delivery solutions for whole tissue replacement or localized regeneration stimulation of wounded tendons. *In vivo* applications in rabbit models have preliminarily confirmed safety of such cell types	In preclinical works, FE002-Ten cells were characterized by a lifespan of 12–15 passages Recommended passages for therapeutic applications are P6 (cell therapies) to P8 (cell-based cell-free formulations) When using the same isolation and culture methods as described for FPCs, adult tenocytes are generally characterized by a lifespan of 7–8 passages	Grognuz et al., 2016a,b Aeberhard et al., 2019 Grognuz et al., 2019
Human cartilage FPCs (e.g., FE002-Cart.Art cell type)	Optimal homogeneity, phenotypic plasticity, and chondrogenic potential have been demonstrated for such cell types, whereas application in caprine models for articular reconstruction has yielded preliminary evidence of safety. Detailed investigation of biochemical and biomechanical parameters of extracellular matrix deposition were performed using such cell types	In preclinical works, FE002-Cart.Art cells were characterized by a lifespan of 10-12 passages, whereas optimal functionality (i.e., ECM generation) was confirmed up to P5 Recommended passages for therapeutic applications are P5 (cell therapies) to P8 (cell-based cell-free formulations) When using the same isolation and culture methods as described for FPCs, adult chondrocytes are generally characterized by a lifespan of 6–8 passages	Quintin et al., 2010 Darwiche et al., 2012 Broguiere et al., 2016 Studer et al., 2017 Cavalli et al., 2018 Li et al., 2020
Human bone FPCs (e.g., FE002-Bone cell type)	Detailed investigation of phenotype modulation and matrix production activities were performed on such cell types, providing extensive insights on the multiple parameters within optimization of skeletal tissue engineering. Murine and rat models have demonstrated safety of application of such cell types	In preclinical works, bone FPCs were studied up to P8–P9 Recommended passages for therapeutic applications are P5 (cell therapies) to P7 (cell-based cell-free formulations)	Montjovent et al., 2004, 2007, 2008, 2009 Hausherr et al., 2017, 2018
Human muscle FPCs (e.g., FE002-Mu cell type)	High interest for applications in tissue reconstruction was evidenced for such cell types, whereas application in murine models has demonstrated safety and absence of immunogenicity for such cell types	In preclinical works, muscle FPCs were studied up to P4–P5	Hirt-Burri et al., 2008a Laurent et al., 2020c
Human intervertebral disc FPCs (e.g., FE002-Disc cell type)	*In vitro* characterization has allowed to establish the tangible potential of such sources for application in skeletal tissue engineering and amelioration of patient quality of life	In preclinical works, intervertebral disc FPCs were studied up to P4–P6	Quintin et al., 2009, 2010
Human lung FPCs (e.g., FE002-Lu cell type)	Such cell sources were studied and benchmarked with currently used biotechnological cellular substrates (e.g., MRC-5), demonstrating high potential for implementation in industrial workflows with augmented safety, consistency, stability, and output. Therapeutic exploitation of anti-inflammatory properties is considered	In preclinical works, FE002-Lu cells were studied up to P20	NA
Ovine FPCs (e.g., AG001-AG005 cell types)	Combination of ovine FPC banking and biotechnological processing has demonstrated the potential for stabilization of tremendous healing stimulation properties and application thereof for topical regenerative effects. Extensive *in vitro* lifespans and high consistency were demonstrated for various primary ovine FPC types, constituting tangible advantages for biological product supply chain sustainability	In preclinical works, ovine FPCs were studied up to P40	Lapp et al., 2013
Equine FPCs (e.g., ED001-ED002 cell types)	The simultaneous multi-organ harvest workflow adopted for human fetal donations was conceptually confirmed and experimentally validated using equine fetal tissues. Subsequent characterization and therapeutic applications of equine FPC therapies have demonstrated high similarities with human regenerative medicine and further broaden the potential therapeutic applications of primary FPC banking	In preclinical works, equine FPCs were studied up to P10	Laurent et al., 2020b

*Specific cell type lifespan characteristics were included, expressed as passages (see Supplementary Material). Data succinctly summarize primary and secondary published works, for which selected references are provided. Due to the renewal of regulatory frameworks and successive adaptations of the Transplantation Programs throughout the years, data accumulated over two decades was generated with primary FPC types isolated from different donations. Unification and standardization of FPC clinical use was operated after processing of the FE002 fetal donation in 2009. The overarching conclusions are the high consistency, extensive banking potential, and proven safety of various primary FPC types of mammalian origin. NA, Non-Applicable.*

## Classic Currents of Thought: Scarcity and Pooling of Therapeutic Cell Sources

In human organ transplantation, the relative scarcity of high therapeutic value biological materials often requires compromise, while maintaining adequate safety and quality standards (Glantz et al., 2008). In the case of blood banks for medical transfusion or industrial-scale manufacturing of human platelet lysate (HPL) and fetal bovine serum (FBS), pooling of multiple donor samples is necessary to achieve the required lot size to produce coherent deliverable quantities after adequate safety and quality testing is performed. Similarly, production of homogenized cell pools for industrially commercialized therapeutic products (e.g., pooled neonatal foreskin keratinocytes) assumes the integration of many variables and potentially heterogeneous components, albeit meeting the specifications for lot qualification and liberation, achieved due to large numbers of donors. Such practices and related technical considerations are well accepted and detailed in pharmacopeia sections on blood-related products, for example. Considerable advantages of focusing efforts on a single donor yielding homogenously derived cell sources enable the abolition of the variability mentioned above, while enabling extensive and rational testing of biological materials. Indeed, screen-testing of donors for pools is then replaced by extensive safety testing of the mother-donor in the Fetal Transplantation Program, followed by routine testing of cell production lots, inherently implemented in GMP workflows, resulting in relatively low overall normalized costs. The consistency, robustness, and extensive cellular expansion capacities within FPC biobanks allow maximal characterization and standardization of biological substrate variables. These crucial aspects were most helpful in the early route to such optimized sources for vaccine or recombinant protein production by the pharmaceutical industry (Applegate et al., 2010). Additionally, optimal conservation and persistence of cellular characteristics throughout whole-cell bioprocessing and maintenance of extensive *in vitro* lifespans negate the necessity of primary cell immortalization into cell lines, thereby minimizing artificial manipulation of the biological materials (Applegate et al., 2009). Low heterogeneity exists between different fetal organ donations and between different samples consistently processed from the same biopsy (Quintin et al., 2007). Optimal consistency in cellular expansion parameters and endpoint cell yields may be achieved, as FPCs do not rely on growth factor supplementation for phenotypic modulation. A paradigm shift toward the replacement of pooled biological materials by cultured FPCs would surely result in optimized availability and affordability of therapeutic products or biotechnological substrates, while maximizing both consistency and safety, due to the numerous relative advantages of FPC biobanking, as described hereafter.

## Swiss Fetal Transplantation Programs

**FIGURE 1:**
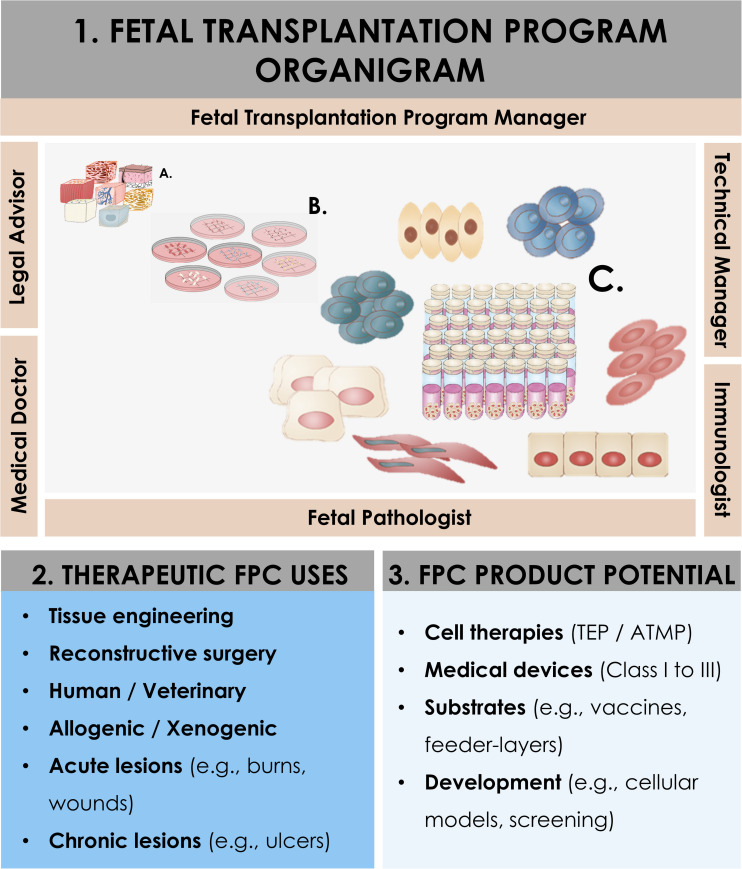
Schematic overview of the components and ramifications of a Fetal Transplantation Program and of primary FPC banking in general, assorted to potential applications and benefits in view of product development. The high core-value is created throughout adequate biopsy procurement, bioprocessing thereof, and establishment of homogenous PCBs of primary FPC types. Essential multidisciplinary building blocks of a human Fetal Transplantation Program comprise complementary expertise and experience, mutualized between the Program Manager (i.e., establishment and coordination of the Program, usually a pharmacist or biologist with extensive experience in tissue processing and cell banking for optimized cell source selection and technical specifications establishment), the Legal Advisor (i.e., interpretation of regulatory frameworks for transplantation practice and therapeutic product use, design and validation of the Program within local and national laws, and regulations on research and medicine), the Technical Manager (i.e., oversight of the bioprocessing and cell banking steps, usually a biologist or senior laboratory technician with extensive experience in tissue processing and cell banking), the Medical Doctor (i.e., experienced gynecologist, performs donor identification, screening, consent obtention, and donation procurement, preferably from a secondary independent hospital), the Fetal Pathologist (i.e., oversight of coded autopsy, preferably experienced in fetal histopathology), and the Immunologist (i.e., pathogen screening of mother-donor biological samples and of established cell banks). A defined organigram enables optimal anonymous traceability within the information flow. Iterative validation steps ensure optimal quality and safety of all processed materials. Pathology and serology reports are evaluated to confirm requirement fulfillment and admissibility of the donor in the Program. Established PCBs are quarantined until the three-month bloodwork results exclude seroconversion of the donor for the target pathogens. Potential applications of banked FPC types are diverse, comprising cell therapy or medical device development for arrays of soft-tissue and musculoskeletal acute and chronic affections (i.e., human and veterinary), *in vitro* fundamental research, and industrial biotechnological manufacturing processes (e.g., viral vaccine production, feeder-layer roles).

Usefulness and adequacy of Fetal Transplantation Programs are most easily demonstrable, and the utilization of robust FPC banks may contribute to the alleviation of the constant organ transplant demand or shortages. The practical design of optimal workflows for cell source selection and processing is paramount when developing cell therapy, tissue bioengineering, or cell-based products. Along with biological material homogeneity, consistency, and robustness, documented traceability and quality also ensure safety and efficacy for clinical applications (Kent and Pfeffer, 2006; Pfeffer and Kent, 2006). Optimization must, therefore, be undertaken for the identification of cell sources, material procurement, and subsequent processing. Transplantation Programs are highly regulated and adaptable frameworks optimally suited for such exhaustive and descriptive activities. Swiss FPC Transplantation Programs were devised in the early 1990s in Lausanne to establish cell banking of primary FPC types after regulated voluntary pregnancy terminations and subsequent organ donations (Applegate et al., 2013). Initially registered in 1991 and reorganized in 2007, the successive Transplantation Programs remain regulated by Swiss federal laws, pertaining to organ transplant procedures, and are registered with the Swiss National therapeutic products agency (i.e., Swissmedic, Bern, Switzerland). Key stakeholders in the Program collaboratively pool complementary professional expertise and capabilities to fulfill respective duties and ensure adequate compartmentalization (Figure 1). Adequate documentation enables appropriate Program validation and follow-up, comprising technical specifications, fetal biobank regulations, and mandatory license documents. Highly regulated and sequentially defined voluntary and therapeutic pregnancy interruptions serve as the operating base for mother-donor recruitment. Regulatory vetting and GMP constraints relative to traceable tissue procurement, testing, and bioprocessing favor an up-stream medical and serological testing approach (i.e., repeated bloodwork for HIV-1/2, HTLV-1/2, hCMV, EBV, HHV-6/7/8, HSV, HBV, HCV, HPV, West Nile virus, syphilis) of mother-donors for inclusion in the Program, positively impacting long-term testing costs (Supplementary Figure 1; Quintin et al., 2007; Applegate et al., 2013). Practically, optimized workflows and specifications eventually enabled traceable simultaneous isolation of various FPC types (i.e., FPCs isolated from fetal tissues such as skin, cartilage, tendon, bone, muscle, intervertebral disc, lung) from a single fetal organ donation (i.e., codename FE002, 2009) for rapid and efficient PCB establishment and subsequent industrial GMP processing (Laurent et al., 2020e). Specific bioprocessing methodologies enable safe and sustained use of original cell sources for extended periods, as adequate testing implementation ensures maximal safety of the end-products or substrates (De Buys Roessingh et al., 2015). One single qualifying fetal organ donation, yielding specific tissue biopsies, is sufficient for the derivation of multi-tiered cryopreserved cell stocks, which may be preserved for decades, minimizing the need for multiple organ donations, ultimately lowering constraint levels related to timeframes and costs.

## Primary FPCs: Strong Scientific and Medical Innovation Background

### Historical Use of FPCs or Embryonic Cell Types and Cell Lines

Fetal and embryonic cells have been extensively used throughout history in the biomedical industry, starting back in the 1930s with the continuous development of numerous vaccines (e.g., chickenpox, Ebola, hepatitis A, HIV, influenza, Japanese encephalitis, polio, rabies, rubella, and smallpox), which are still currently in use (Jacobs et al., 1970; Reisinger et al., 2009; Applegate et al., 2013, 2017). A Nobel Prize in medicine was given in 1954 for the polio vaccine, developed using human fetal cell cultures. Such industrial uses demonstrate the *quasi*-universal applicability of fetal cells as substrates in therapeutic product manufacturing, providing excellent in-use safety and stability (Hayflick et al., 1962; Jacobs et al., 1970; Zimmerman, 2004; Olshansky and Hayflick, 2017). Specific human embryonic/fetal tissues and/or animal biopsies led to the establishment of well-known cell types or cell lines (e.g., HEK-293, MDCK, MRC-5, PER.C6, and WI-38/CCL-75) (Palache et al., 1997; Zimmerman, 2004). Early therapeutic use of fetal tissue or derived FPCs focused on neurology (e.g., Huntington’s or Parkinson’s disease, strokes, spinal cord injuries) (Freeman, 1997; Clarkson, 2001; Rosser and Dunnett, 2003; Reier, 2004; Savitz et al., 2004). Fetal hepatic cells were studied and transplanted to manage severe hematological disorders, immunodeficiencies, liver failure, diabetes, and congenital metabolic disorders (Touraine et al., 1993; Gridelli et al., 2012; Montanucci et al., 2013; Cardinale et al., 2014). In clinical settings, fetal hepatocyte infusions have been performed in more than 30 patients so far in view of alleviating transplant shortages, with promising results yielded mostly by one research group in India (Habibullah et al., 1994; Khan et al., 2010).

### Specific Characteristics and Therapeutic Potential of FPCs

**FIGURE 2:**
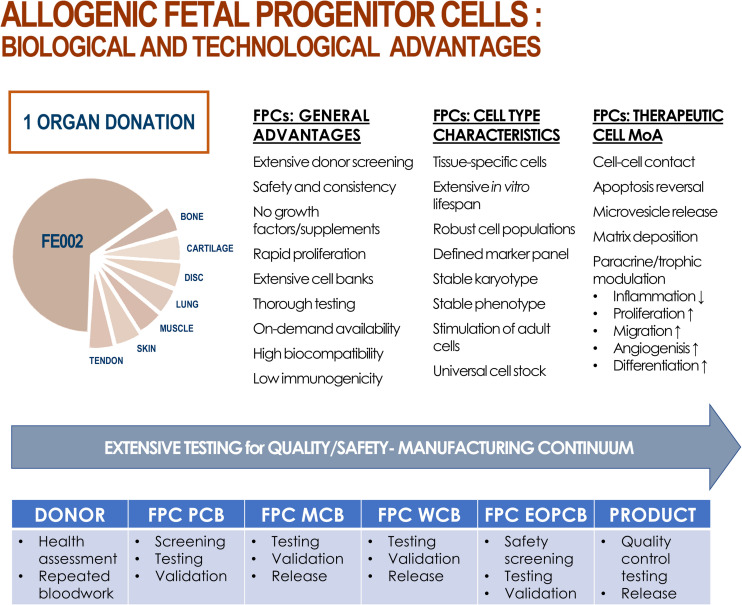
Summary of biological and technological advantages of processing and using FPCs as therapeutic agents, production intermediates, or substrates. From one single fetal organ donation (i.e., FE002, 2009), various tissue samples (e.g., bone, cartilage, intervertebral disc, lung, muscle, skin, tendon) were bioprocessed for FPC isolation using both enzymatic and non-enzymatic methods. Inherent technical and clinical advantages are attributed to FPCs. Various proposed mechanisms of action (MoA) of allogenic FPCs are summarized. Optimized and consistent tissue procurement, cell isolation, and biobanking workflows allow for thorough testing throughout the manufacturing continuum, ensuring quality and safety of end-products.

Fetal wound healing before mid-gestational stages is specifically and characteristically orchestrated, leading to regeneration without scar tissue formation in several organs and structures (e.g., skin, bone, cartilage, tendon) (Adzick and Longaker, 1992; Longaker et al., 1992; Namba et al., 1998; Beredjiklian et al., 2003; Bullard et al., 2003; Dang et al., 2003; Favata et al., 2006; Rodrigues et al., 2019). Cultured FPCs isolated after nine weeks of gestation are pre-terminally differentiated, possessing finite high expansion capacities, and scarless regeneration stimulation potentials, while presenting low risks of immunogenicity or tumorigenicity after transplantation (Figure 2; Doyle and Griffiths, 1998; Quintin et al., 2007; Markeson et al., 2015; Laurent et al., 2020d). Differential gene expression (e.g., genes coding for TGF-β2, BMP-6, GDF-10, midkine, or pleiotrophin) and related proteomic fingerprints may explain specific healing patterns mediated by adult cells and FPCs (Hirt-Burri et al., 2008b). As early descendants of stem cells, FPCs are found in diverse developed tissues (e.g., skin, intestine, blood system, brain), mediating tissue homeostasis and repair (Nakatomi et al., 2002). Along with the absence of self-renewal capacity, relatively restricted potency distinguishes FPCs and stem cells, as FPCs are reportedly unipotent or oligopotent, providing relatively superior phenotypic stability. Technically, FPCs are independent of growth factor supplementation or presence of cellular feeder-layers for *in vitro* cultures (Asahara et al., 1997; Seaberg and van der Kooy, 2003). This specific inherent advantage over undifferentiated MSCs or induced pluripotent stem cells (iPSC) primarily benefits consistency in manufacturing and industrial scale-up processes (Doyle and Griffiths, 1998; Ramelet et al., 2009; Zuliani et al., 2013; Tan et al., 2014; Lee et al., 2020). Constraints on production timelines and economic factors additionally favor the use of low-maintenance and robust cell types such as primary FPCs.

### Immune Privileges of FPCs

Fetal progenitor cells are pre-immunocompetent and fail in eliciting immunological responses due to the lack of post-thymic T-lymphocytes in the first 13 gestational weeks (Gabbianelli et al., 1990; Crombleholme et al., 1991). Major histo-compatibility complex (MHC) antigen expression during fetal development is organ- and gestational age-specific (Foglia et al., 1986). Primary FPCs generally lack MHC class II proteins (e.g., HLA-DP, DQ, DR) and exhibit relatively low levels of MHC class I counterparts (e.g., HLA-A, B, C), approaching cell surface marker panels characterizing MSCs or neonatal foreskin keratinocytes, for example (Tsujisaki et al., 1987; Streit and Braathen, 2000; Le Blanc et al., 2003; Grognuz et al., 2016b). Specific fetal tissues were shown to express HLA-G, a known mediator of tolerogenic effects (Piccinni, 2010; Deschaseaux et al., 2011). Fetal progenitor cells therefore evade immune responses, possibly through immune-modulation and inhibition of TCD8^+^ lymphocyte proliferation (Bartholomew et al., 2002; Le Blanc et al., 2003). Absence during normal human gestation of an immune reaction, despite *in utero* recognition of paternal HLA-C markers and modulatory effects of HLA-G on lymphocytic activity, additionally characterize the particular immune status of fetal tissues and FPCs (Rouas-Freiss et al., 1997; Ober, 1998; Carosella et al., 2008; Piccinni, 2010).

### Technical Simplicity, Stability, and Robustness of FPCs

**FIGURE 3:**
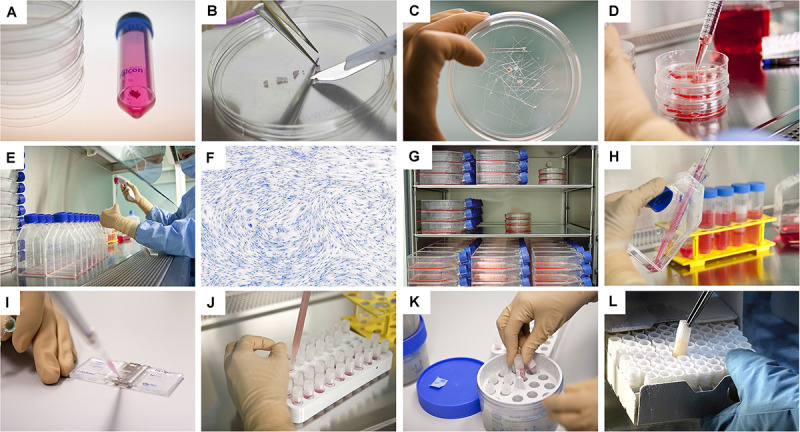
Overview of the simple and standardized mechanical procedure for primary FPC type isolation from organ donation tissue biopsies and Parental Cell Bank establishment. Highly similar simultaneous processing of multiple tissues yielded by one organ donation enables maximal consistency throughout progeny cell populations. **(A)** Individual anonymized tissue biopsies are provided by the pathology department in transport medium. Each specific tissue of interest is separately conditioned. Fetal skin is used as an example herein. **(B)** Tissue biopsies are further processed into small fragments. **(C)** Tissue fragments are minced and placed within a checkboard pattern created on the culture surface by scoring with a sterile scalpel. **(D)** Cultures are initially fed with small amounts of growth medium in order to avoid early flotation of fragments. **(E)** Adherent cells are further expanded in culture flasks. **(F)** Cells are regularly microscopically assessed to verify adequate morphology or growth and to exclude contamination. **(G)** Multiple FPC types are simultaneously culture-expanded in humidified incubators set at 37°C under 80% relative humidity and 5% CO_2_. **(H)** Confluent cells are harvested by trypsin detachment and pooled. **(I)** Total and viable relative cell counts are determined by microscopic enumeration using Trypan blue exclusion dye. **(J)** Cells are resuspended in a cryopreservation solution (i.e., DMEM, FBS, DMSO) and homogenously dispensed in individual cryovials (i.e., 10^6^–10^7^ viable cells/vial). **(K)** Vials are transferred to controlled-rate freezing devices (e.g., Mr. Frosty^TM^ or CoolCells^®^) and placed in ultra-low temperature freezers (i.e., −80°C) overnight. **(L)** Cryovials are then transferred to Dewar storage tanks in the gaseous phase of liquid nitrogen for long-term storage. Some technical limitations in large-scale cell bank manufacturing are outlined and must be the object of continuous optimization. Such limits comprise, without being limited to, operator-related cell quantification, relatively important occupied volumes in conventional incubators with limited airflow and oxygenation, or relative contamination risks (e.g., open vessels for cryopreservation).

**FIGURE 4:**
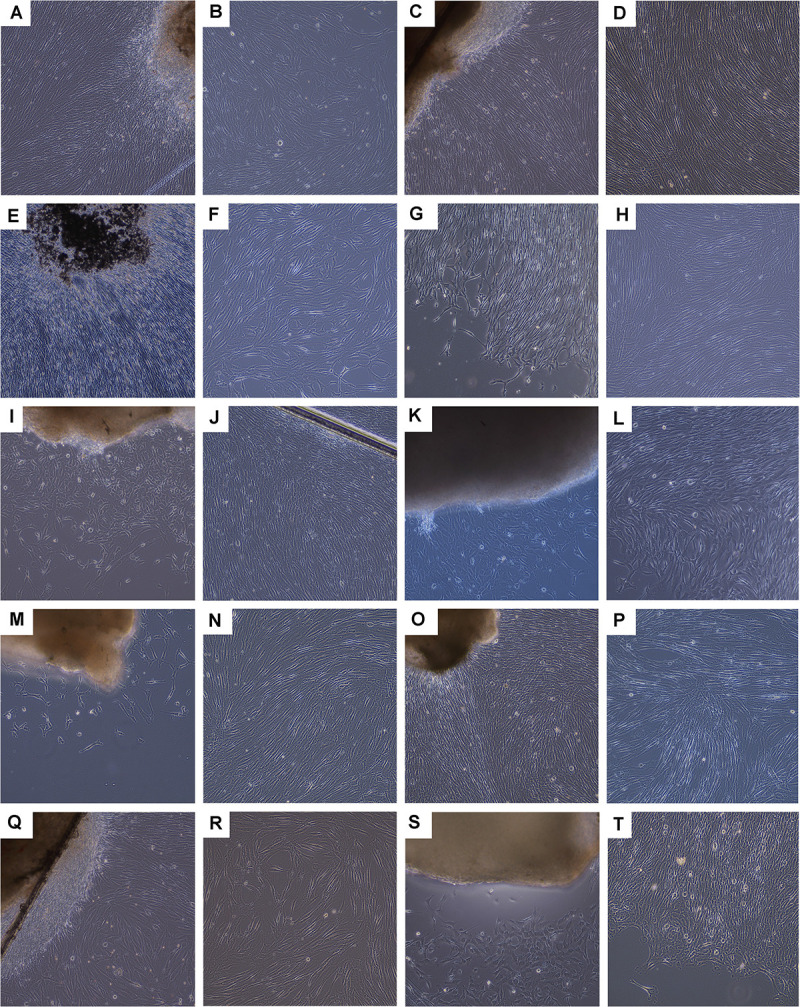
Photographic imaging of culture initiation and culture-expansion steps performed following a fetal organ donation within updated legislative frameworks (i.e., post-2007). Various tissue biopsies were procured from the same organ donation (i.e., FE002, 2009) and simultaneously differentially processed following enzymatic and non-enzymatic methodologies. Pictures were obtained under 100X optical magnification on a phase contrast microscope and represent the non-enzymatically isolated primary FPC types. **(A,B)** Ventral skin with emitting dermal FPCs (i.e., FE002-SK1 cell type, P0). **(C,D)** Dorsal skin with emitting dermal FPCs (i.e., FE002-SK2 cell type, P0) and confluent cells at P2. **(E,F)** Tendon tissue with emitting tendon FPCs (i.e., FE002-Ten cell type, P0). **(G,H)** Articular cartilage with emitting cartilage FPCs (i.e., FE002-Cart.Art cell type, P0) and confluent cells at P2. **(I,J)** Cartilage tissue with emitting cartilage FPCs (i.e., FE002-Cart cell type, P0). **(K,L)** Bone tissue with emitting bone FPCs (i.e., FE002-Bone cell type, P0). **(M,N)** Intervertebral disc tissue with emitting disc FPCs (i.e., FE002-Disc cell type, P0) and confluent cells at P1. **(O,P)** Lung tissue with emitting lung FPCs (i.e., FE002-Lu cell type, P0) and confluent cells at P1. **(Q,R)** Muscle tissue with emitting muscle FPCs (i.e., FE002-Mu cell type, P0) and expanding cells at P2. **(S,T)** Connective tissue with emitting connective tissue FPCs (i.e., FE002-CT cell type, P0). For higher magnification, see Supplementary Figure S5.

**FIGURE 5:**
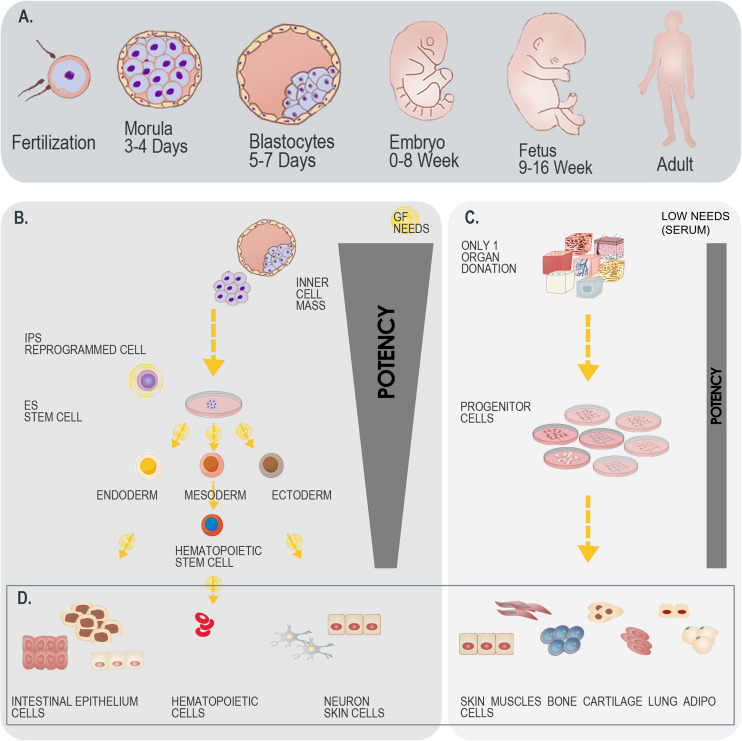
Differential overview highlighting the similarities and differences between stem cells and primary FPC types. **(A)** Schematic representation of developmental stages within the human biological continuum, assorted to classes of cells to potentially be isolated. **(B)** Schematic representation of the isolation and culture-expansion of stem cells from blastocytes. Such cell types may serve for subsequent processing and generation of various stem cell types (e.g., iPSCs). **(C)** Schematic representation of the simultaneous isolation and culture-expansion of primary FPCs. Such procedures are relatively simpler and more robust than when working with stem cells, as a single organ donation enables differential isolation of several tissue-specific cell types, without the resort to growth factor (GF) cocktails in culture-expansion and maintenance steps, which largely and positively impact the consistency of progeny cellular materials. **(D)** Schematic representation of cellular materials obtained after biopsy processing and cell bank establishment. Differentiated cell types are eventually obtained when using both starting materials (i.e., blastocytes versus fetal tissue organ donations), with specificities to each strategy. A single isolation procedure is necessary when working with blastocytes, whereas differential biochemical manipulation enables generation of various cellular phenotypes maintaining designed relatively restricted potency. A single isolation procedure is equally necessary when working with fetal tissues, whereas standardized parallel processing enables generation of homogenous FPC types, inherently relatively restricted in terms of potency. Overall, while both strategies for therapeutic cell type obtention may be compared, the use of primary FPCs is relatively more robust, may be standardized, is cost-effective and sustainable.

The ability of therapeutic cells to maintain inherent biological characteristics, when isolated *in vitro*, presents considerable potential for tissue engineering. Differential requirements for processing and clinical delivery specifically characterize ESCs, adult MSCs, and FPCs, whereas numerous technical advantages favor the use of the latter (Bhattacharya, 2004; Ostrer et al., 2006; Capes-Davis et al., 2010). Embryonic stem cells can be derived from the blastocyte (i.e., constituted by approximately 100 cells) between zero and two weeks after ovum fertilization. These “immortal” cells require growth factor support in culture or appropriate feeder-layers to sustain growth, potentially introducing inconsistencies in progeny cell populations. Additionally, ethical concerns, propensity toward tumorigenicity, and high potency render the obtention and use of such populations difficult. Embryonic fetal cells can be derived at timepoints between five and eight weeks of gestation (i.e., total size of >10^3^ cells/embryo). Relatively restricted potency compared to ESCs characterizes these populations, but all other disadvantages remain, assorted to onerous culture and maintenance requirements. Fetal tissues (i.e., total size of >10^6^ cell/fetus) exist in the developing organism between weeks number nine and sixteen of the gestational period. Fetal progenitor cells yielded by various fetal tissues are therefore pre-terminally differentiated and present defined tissue-specific properties and behaviors, which are conserved in monolayer *in vitro* cultures. In contrast, MSCs are scarce or difficult to isolate and to purify for obtention of adequate cell populations, are patient-specific because of immunological and safety factors, and therefore necessitate multiple organ donations, whereas culture scale-up is difficult to implement. Legal distinctions categorize work around cellular material existing before and up to eight weeks of gestation, as a federal license is required in Switzerland. Starting at nine weeks of gestation, studies with specific fetal tissue biopsies are regulated under Federal Transplantation Laws, and such tissues are defined as organ donations. Standardized isolation methods for FPCs in defined gestational timeframes yield uniform preliminary cultured populations characterized by homogenous and stable tissue-specific properties, without the need for specific cell-sorting (Figures 2–5 and Supplementary Figures 2–7; Quintin et al., 2007, 2009, 2010). Progeny FPCs are characterized by their relatively high and consistent division potential *in vitro* before reaching senescence due to their relatively longer telomeres (Decary et al., 1997). Therapeutic applications in clinical protocols or product manufacturing workflows in regenerative medicine restrict the use of progeny cell sub-cultures to two thirds of the documented and safety-validated *in vitro* lifespans of specific cell types. Such regulations ensure end-product consistency and maintenance of paramount cellular biological properties, such as cumulative or specific protein content (e.g., MDK, MMP, TGF, TIMP, and VEGF levels), gene expression levels, and bio-stimulatory activities to be assessed *via* quantitative quality controls or functional assays (Vuadens et al., 2003; Quintin et al., 2007). A benefit of using allogenic banked cellular substrates instead of autologous sources is the drastic reduction in availability delays, as off-the-freezer cell therapies or stabilized cell-derivatives may be available upon request. Maximized safety and quality of end-products are demonstrable with banked FPCs, allowing realistic clinical translation, transposition to industrial settings, and commercial implementation in leading markets, well within current regulatory frameworks and sustainable developmental economic burdens (Quintin et al., 2007; Larijani et al., 2015; Marks and Gottlieb, 2018).

### Swiss Multi-Tiered Biobanking Model for Primary FPCs

**FIGURE 6:**
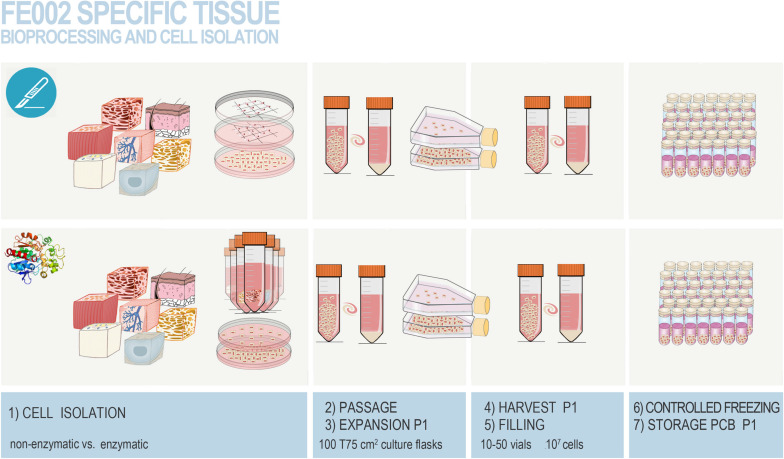
Overview of the simultaneous differential biopsy processing methods devised for the FE002 fetal organ donation in view of adherent FPC culture initiation. The different fetal tissues were simultaneously either submitted to enzymatic or non-enzymatic processing. Individual tissue biopsies from the FE002 donation were procured by the pathology department and further dissected into fragments, providing starting material for both cell isolation methods. All fragments were washed thrice in phosphate buffered saline supplemented with 1% penicillin–streptomycin. **(1)** Fragments were then either appropriately dissected and placed in scored sterile culture dishes (i.e., non-enzymatic workflow) or subjected to appropriate trypsin digestion (i.e., enzymatic workflow) before plating in culture dishes. Sufficient amounts of seeded culture vessels were prepared for each individual tissue type and both cell isolation methods. Cells and tissue fragments were cultured in Dulbecco’s Modified Eagle Medium (DMEM) supplemented with 10% clinical-grade fetal bovine serum (FBS). Cultures were incubated in a 37°C humidified incubator under 5% CO_2_ and the growth medium was renewed every other day. **(2)** After rapid cell emission or free proliferation, preliminary cultures (i.e., P0) were harvested by trypsinization after attaining 90 % confluency. **(3)** Cells were then enumerated and used to seed sufficient amounts of vented cell culture flasks for further expansion (i.e., P1). Culture medium was thereafter composed of DMEM, FBS, and additional L-glutamine. **(4,5)** Once optimal banking confluency was reached, cells at P1 were harvested, enumerated, and conditioned in individual 1 mL aliquots in a DMSO-based cryopreservation solution for long-term storage. **(6,7)** Cryovials were frozen following a controlled rate and were transferred to the vapor phase of separate level-alarm-fitted locked Dewar storage tanks to constitute the Parental Cell Banks. Figure adapted with permission from Laurent et al. (2020e).

**FIGURE 7:**
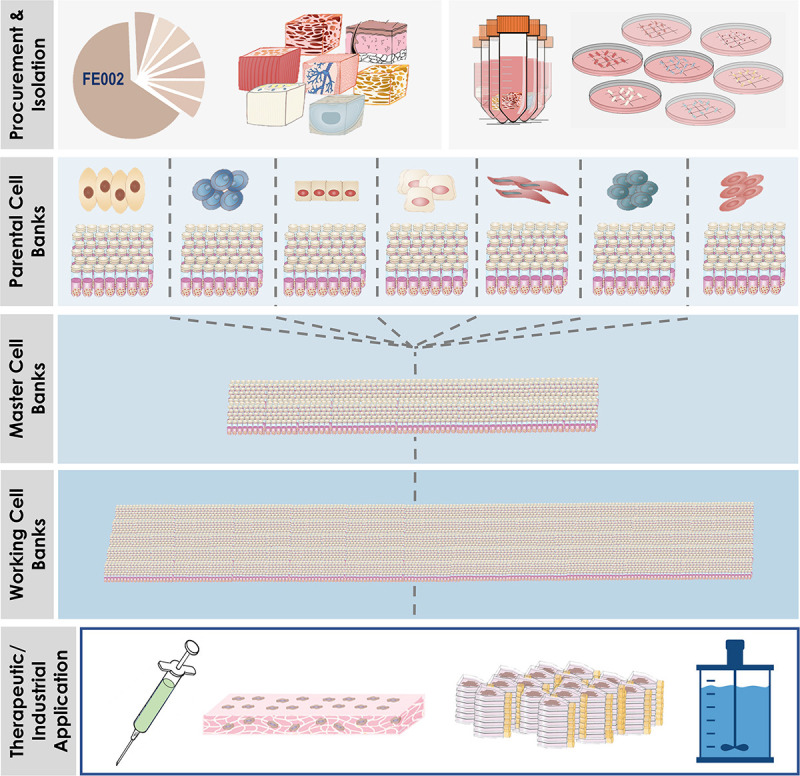
Overview of simultaneous differential establishment of various primary FPC types following specific processing of biopsies from the same single organ donation (i.e., FE002, 2009). Procurement of the donation and micro-dissection enabled the specific tissue processing workflows to be implemented (i.e., enzymatic or non-enzymatic adherent cell culture initiation). Following the establishment of the tissue-specific FPC types, multi-tiered cell banking was performed in parallel for each specific cell type. Materials from Working Cell Banks were then used for diversified applications, which comprised or may comprise therapeutic live-cell product manufacture, use of FPCs or cellular materials as feeder-layers or culture supplements, and use of FPCs as substrates for biotechnological applications (e.g., viral vaccine production).

**FIGURE 8:**
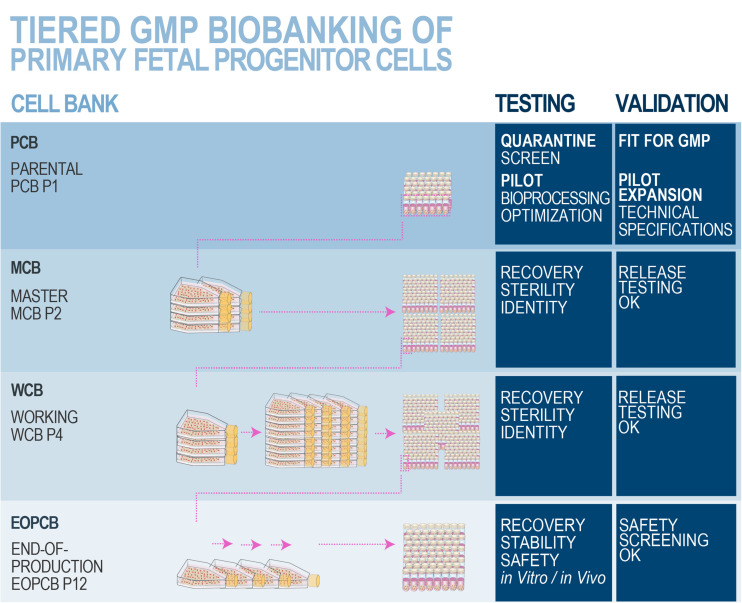
Schematic overview of optimized and standardized multi-tiered cell banking workflows for primary FPCs. *In vitro* optimization steps performed during the pilot study may comprise serum lot choice, culture surface, and brand comparison, in order to maximize cell viabilities and population doubling values within minimal timeframes, obtaining the highest endpoint cell yields and best efficiency of manufacturing. Specific product release and characterization testing for the manufactured cell bank lots may comprise cell growth, isoenzyme testing to confirm cell type origin, DNA fingerprinting of the cell type, qualification/testing for sterility, specific testing for the absence of endotoxins, mycoplasma, viral contaminants (e.g., picornavirus, orthomyxovirus, paramyxovirus, adenovirus, reovirus, West Nile virus, BPyV, HuPyV, HPV, HBoV, WUPyV, KIPyV, EBV, HAV, HBV, HCV, hCMV, HIV-1, HIV-2, HTLV-1, HTLV-2, HHV-6, HHV-7, HHV-8, SV40, and B19 parovirus), evaluation of reverse transcriptase activity, and quantitative transmission electron microscopy (TEM) of cell sections for the detection of viruses, virus-like particles, mycoplasma, yeasts, fungi, bacteria (i.e., ≥200 cell profiles). Safety assessments may be performed on EOPCB materials and comprise *in vivo* tumorigenicity assays and karyology studies. Nomenclature for correlated bank tier and passage numbers is provided here as an example, as it has been validated for dermal FPCs (i.e., FE002-SK2 cell type). The devised technical specifications, testing, and validation strategies are optimally adapted for banking FPCs, due to the inherent high robustness, consistency, and stability of the considered cellular materials. Industrial transposition towards GMP production is therefore tangibly attained with such materials, as extensive multi-tiered cryopreserved cell banks may be rapidly and efficiently established. Figure adapted with permission from Laurent et al. (2020e).

Optimal stability and consistency of FPCs derived from one single organ donation present a vast potential toward scalable and extensive biobanking, while following stringent safety- and quality-driven regulations for therapeutic product manufacturing (Abbasalizadeh and Baharvand, 2013; Hunsberger et al., 2015; Laurent et al., 2020e,g). Albeit finite, *in vitro* lifespans and expansion potentials of primary FPCs are sufficient for industrial-scale GMP manufacturing with minimal processing requirements. Standardized multi-tiered cell banking model establishment (i.e., sub-tiering cryopreserved cell stocks in Parental, Master, Working, and End of Production Cell Banks, PCB-MCB-WCB-EOPCB, with tier nomenclature based on *in vitro* passages) allows for efficient constitution, transposition, and utilization of consistent biological sources of high therapeutic value (Figures 6, 7; De Buys Roessingh et al., 2013; Laurent et al., 2020e). Rapid establishment of such cryopreserved materials allows for *quasi*-infinite research and development, as each FPC type from the original organ donation may be valorized to provide >10^7^–10^9^ product doses. Local applications (e.g., skin, tendon, or cartilage tissue repair) of relatively small doses of cells or derivative equivalents (i.e., 5 × 10^5^–10^6^ units, cell type-specific) are optimal and preferable to systemic delivery, as they allow sparing use of biological materials, compared to alternative therapeutic cell sources (e.g., 10^8^ cells/dose for MSCs or 10^9^ cells/dose for pluripotent stem cells) (Hohlfeld et al., 2005; Pigeau et al., 2018; Pittenger et al., 2019). At the same time, safety testing and quality controls are easily implemented throughout bioprocessing workflows (Figure 8; Quintin et al., 2007). Derivation of multiple FPC types from a single organ donation and the development of robust analytical technologies drastically simplify screening and testing processes during manufacturing (e.g., tests for sterility, isoenzyme typing, mycoplasma, viruses, prions, endotoxins, virus-like particles, retroviral activity, fungi, yeasts, bacteria, and tumorigenesis assays) (Applegate et al., 2009). Maximized safety, efficiency, and optimized industrial manufacturing schemes cost-enable innovative therapeutic developmental research and ensure on-demand availability of end-products (Haack-Sørensen and Kastrup, 2011; Abbasalizadeh et al., 2017; Pigeau et al., 2018; Hunt, 2019).

### Human Dermal FPCs (e.g., FE002-SK1, FE002-SK2 Cell Types)

#### Cell Therapies for Cutaneous Regenerative Medicine

Sub-optimal pharmacotherapeutic management of severe and complex cutaneous affections and complications (e.g., chronic ulcers, burns, donor-site wounds) has prompted the development of numerous skin graft solutions (e.g., amniotic membrane, cadaver grafts, fish skin), innovative bioengineered cellular therapy solutions (e.g., cultured autografts), or autologous and allogenic cell-based products (e.g., Allox^®^, Apligraf^®^, Epicel^®^, Lyphoderm^®^, OrCel^®^, ReCell^®^, TransCyte^TM^) that complement surgical care and support tissue structural integrity and functional recovery (Lukish et al., 2001; Limat and Hunziker, 2002; Kumar et al., 2004; Amani et al., 2006; Hartmann et al., 2007; Zaulyanov and Kirsner, 2007; Akita et al., 2008; Hirt-Burri et al., 2008b; Guerid et al., 2013; Zuliani et al., 2013; Malhotra and Jain, 2014; Tan et al., 2014; Debels et al., 2015; Akershoek et al., 2016; Abdel-Sayed et al., 2019b; Lima-Junior et al., 2019; Momeni et al., 2019; Climov et al., 2020). Further optimization of biological starting materials for such advanced solutions may primarily benefit from banked dermal FPCs (e.g., FE002-SK2 cell type), which have displayed clinical benefits in topically managing complex dermatological conditions, such as actinic dermatitis, eczema, or psoriasis. Cell-laden bioengineered constructs and cell-derivative formulations using dermal FPCs present potent therapeutic results (Hirt-Burri et al., 2011; Moore et al., 2018; Lorant et al., 2019; Poinas et al., 2019). Adapted pharmaceutical forms and delivery scaffolds are moldable and biocompatible with wounded tissues and therapeutic cells, providing optimal physical characteristics (e.g., porosity and mechanical stability). These scaffolds also allow the development of cell contraction forces and homogenous distribution of therapeutic biological substrates. Possible matrices comprise nylon mesh, silicone, collagen (i.e., bovine, equine, or porcine), polyglycolic acid, or hyaluronic acid (HA). Additionally, synergistic *in vitro* effects are yielded by combining polycationic dendrimers and collagen matrices, providing potent anti-microbial effects coupled with keratinocyte migration stimulation and direct angiogenic effects (Abdel-Sayed et al., 2016). Further optimization of biological material processing will enable the transition from off-the-freezer to off-the-shelf therapies, with shortened production and availability delays, simplified logistics, and maintained therapeutic potential (Hunsberger et al., 2015; Li and Maitz, 2018). Probable therapeutic mechanisms of action of FPCs comprise paracrine signaling, with the release of well-proportioned arrays of growth factors or cytokines, and deposition of extracellular matrix (ECM) proteins in wounded environments (Spiekstra et al., 2007). Modulation of inflammation, cell migration and proliferation, immune system, and angiogenesis induction then leads to facilitated tissue repair or regeneration (Werner et al., 2007; Barrientos et al., 2008; Providence et al., 2008; Wojtowicz et al., 2014; Varkey et al., 2015). Due to the robustness of dermal FPCs, many alternative applications are envisioned for *in vitro* standardized models of screening assays or biotechnological manufacturing processes (e.g., feeder-layers, growth supplements for keratinocytes or MSCs, therapeutic cell-free extracts) (Hirt-Burri et al., 2011; Krähenbühl et al., 2015; Patrulea et al., 2015, 2019; Laurent et al., 2020e,i).

#### Swiss Tools for Cutaneous Regeneration: Progenitor Biological Bandages

**FIGURE 9:**
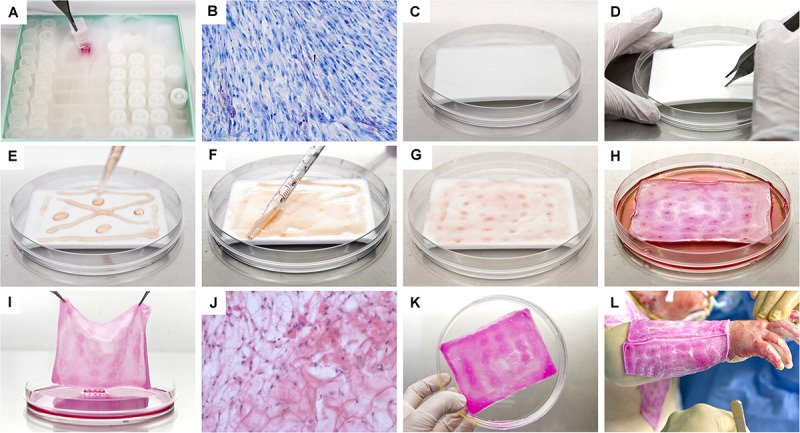
Photographic illustrations providing an overview of the supply chain and manufacturing steps necessary for the preparation of Progenitor Biological Bandages (PBBs), following GMP standards, for clinical application in the Lausanne Burn Center. **(A)** Upon manufacturing order receipt from the clinic, vials from the dermal FPC Working Cell Bank (i.e., FE002-SK2 WCB, P7-P8) are selected and initiated for therapeutic construct preparation. **(B)** Cell suspensions are thawed and cellular viability is assessed. **(C)** Sufficient amounts of equine collagen scaffolds are procured. **(D)** Scaffolds are pre-conditioned by symmetrical puncture of the whole surface. **(E)** Cells are rinsed and seeded on the scaffolds. **(F)** Cell suspensions are further homogenously distributed over the integral surface of the scaffold, to allow optimal cell colonization and integration. **(G)** Seeded scaffolds are further processed to allow uptake of cell suspensions. **(H)** Constructs are incubated for 24–48 h at 37°C under 5% CO_2_. **(I)** After incubation, the scaffolds are checked following quality assurance specifications. **(J)** Histological investigation of a cell-seeded construct (i.e., PBB) after snap-freezing and staining with hematoxylin and eosin. **(K)** PBBs are rinsed and delivered to the operating theater in isotherm containers. **(L)** After standard surgical wound care and disinfection, the constructs are applied and subsequently overlaid with bandages to favor wound healing rate acceleration.

**FIGURE 10:**
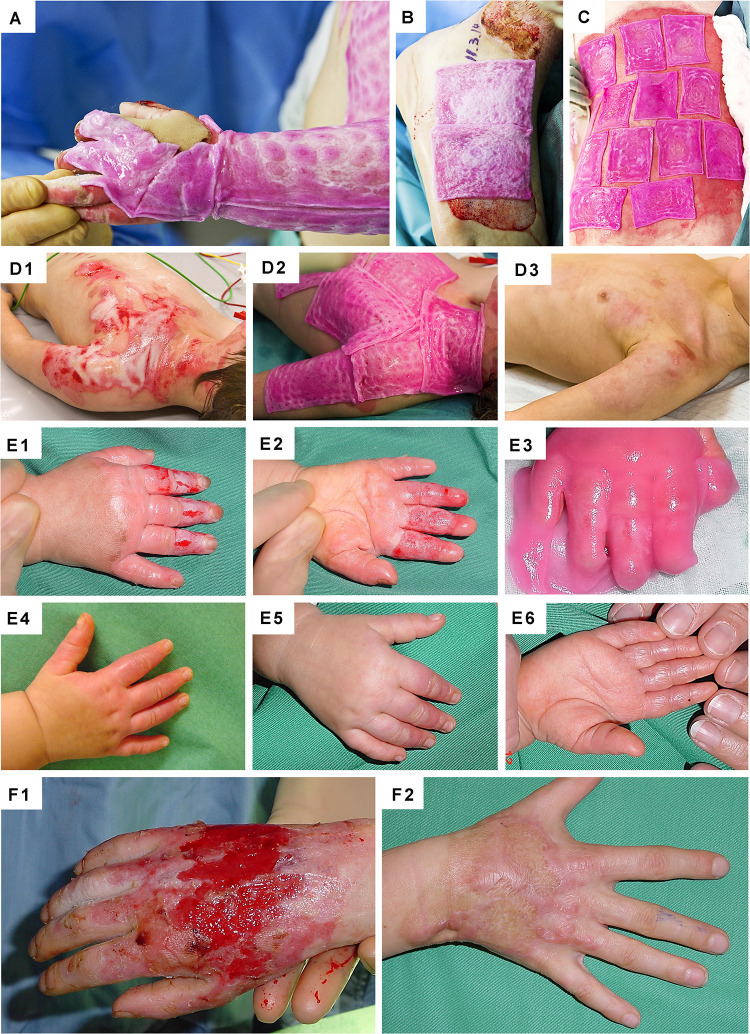
Clinical case-reports illustrating the use and efficacy of Progenitor Biological Bandages for the management of human pediatric burns and donor-site wounds. **(A–C)** Photographic representation of Progenitor Biological Bandages used for primary lesions of a pediatric burn victim and donor-site graft secondary wounds. Unlike skin autografts or synthetic wound coverage solutions, PBBs do not need to be stapled to the patient, as they are simply applied and overlaid with Vaseline gauze before standard bandages are adjusted. **(D1–D3)** Second-degree deep pediatric burn wound (i.e., scalding liquid). Photographic representations of the lesions after early debridement, after PBB application, and after six weeks of treatment. **(E1–E6)** Second and third-degree pediatric burn wound (i.e., scalding liquid). Photographic representations of the lesions after early debridement, after PBB application, and after six weeks of treatment. **(F1–F2)** Second-degree pediatric burn wound (i.e., scalding liquid). Photographic representations of the lesions after early debridement and after ten years during patient long-term follow-up. Figures modified with permission from Hohlfeld et al. (2005) and Laurent et al. (2020a).

**FIGURE 11:**
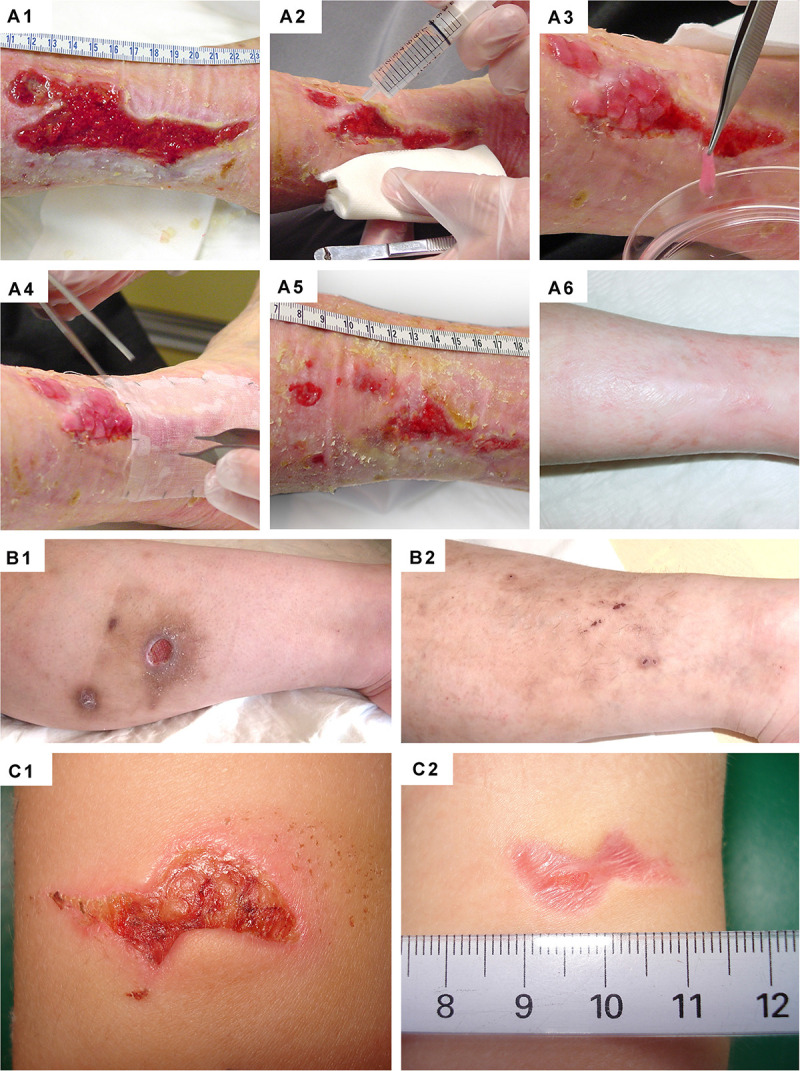
Clinical case-reports highlighting the efficacy of primary FPCs and derivatives thereof for the management of human acute and chronic cutaneous affections. **(A1–A6)** Refractory painful post-thrombotic ulcer lesions were treated weekly with Progenitor Biological Bandages and evolutive photographic representations were acquired at the time of treatment initiation, 11 weeks later, and 15 months later for follow-up. **(B1–B2)** Refractory atypical lower-leg ulcer lesions were treated as for the previous patient, and evolutive photographic representations were acquired at the time of treatment initiation and five weeks later for follow-up. **(C1–C2)** Sharp-force trauma wounds were treated daily with ovine FPC derivatives formulated in a cell-free pharmaceutical cream, and evolutive photographic representations were acquired at the time of treatment initiation and two weeks later. Figures modified with permission from Hirt-Burri et al. (2011) and Lapp et al. (2013).

Progenitor biological bandages (PBB) consist of moldable, single-use, non-invasive bioresorbable wound coverages composed of dermal FPCs yielded by equine collagen scaffolds (9 cm × 12 cm), which are currently GMP-manufactured and clinically delivered on-demand in less than 48 h to the Lausanne Burn Center (Figure 9). Advantages of PBBs comprise a simple and relatively painless one-step application, without staples, providing cost-effective healing promotion within different types of cutaneous lesions (Abdel-Sayed et al., 2019a,b). Such constructs were successfully applied for various cutaneous conditions such as pediatric and adult severe burns, sharp-force trauma wounds, geriatric refractory chronic ulcers, and donor-site wounds, yielding unique reconstructive results (Figures 10, 11; Hohlfeld et al., 2005; Ramelet et al., 2009; De Buys Roessingh et al., 2015). Skin regeneration was achieved extremely rapidly, with the restoration of high elastic properties and improved pigmentation balance, which was without pain, hypertrophy, retraction, inflammation, or the necessity for additional skin grafts. Bioengineered PBB constructs were observed to promote proliferation, adhesion, and migration of endogenous cells, without atrophic skin formation (Ramelet et al., 2009). Over two decades of clinical experience and multicentric studies have shown the safety or beneficial therapeutic effects of dermal FPCs in PBBs, notably within phase I and II clinical trials in Switzerland and Asia (i.e., ClinicalTrials.gov identifiers: NCT02737748 & NCT03624023) (Hohlfeld et al., 2005; Ramelet et al., 2009; Laurent et al., 2020e). In view of further optimization of burn wound or ulcer care in particular, high therapeutic benefits may be gained by stabilizing and formulating active cell-derivative components in pharmaceutical creams, ointments, or gels, as these are used for the maintenance therapy to accelerate wound healing (i.e., potentially scarlessly) after primary wound closure.

### Human Cartilage FPCs (e.g., FE002-Cart, FE002-Cart.Art Cell Types)

#### Cartilage FPCs in Regenerative Medicine

Due to frequent cartilage defects caused by degenerative diseases or excessive wear, cell therapies, cell-based approaches, or combined bioengineered constructs are of high interest for translational medicine applications and predominate developmental efforts (Vrahas et al., 2004; Flanigan et al., 2010; Makris et al., 2015; Carluccio et al., 2020). The avascular and alymphatic nature of cartilage tissues confers relative immune privileges (i.e., isolation from antigen-presenting cells, migratory macrophages, and dendritic cells) and renders allogenic cell therapy approaches possible for tissue regeneration chaperoning (Quintin et al., 2010; Studer et al., 2017). Autologous cartilage cell therapy implementation remains hindered or delayed due to the induction of hypertrophic tissue phenotypes, fibrocartilage formation, high-cost cell expansions, *in vitro* de-differentiation, two-step surgery, donor-site morbidity, and high variability in functional outcomes (Brittberg et al., 1994; Horas et al., 2003; Lu et al., 2006; Katopodi et al., 2009; Vinardell et al., 2012). Differential autologous and allogenic approaches comprise high cellular variability, and related inhomogeneous potency restricts potential therapeutic benefits (Wakitani et al., 2007; Stolzing et al., 2008; Prockop, 2009; Pelttari et al., 2014; Pleumeekers et al., 2014; Steinwachs et al., 2014). Neonatal chondrocytes or cartilage FPCs are optimal candidates for cell therapies, possessing relatively superior chondrogenic potential (i.e., constitutive immature chondrodifferentiation for the latter cell types) than adult chondrocytes (Almqvist et al., 2009; Adkisson et al., 2010a,b; Quintin et al., 2010; Acosta et al., 2011; Darwiche et al., 2012; Dhollander et al., 2012; Cavalli et al., 2018). Fetal progenitor cells also present relatively low hypertrophy marker expression (e.g., type X collagen), possibly due to epigenetic modulations *in vivo* (Zimmermann et al., 2008; Tompkins et al., 2013). Clinical translation of therapeutic cartilage FPCs is appealing due to the potential to consistently treat large numbers of patients (i.e., >10^8^ individual therapies consisting of cell-seeded biocompatible implants following a single fetal organ donation) (Darwiche et al., 2012).

#### Phenotypic Stability, Chondrogenic Potential, and Biomechanics

High phenotypic stability and chondrogenic potential (i.e., elevated sulfated GAG content, Sox9:Scleraxis ratios, *IHH* and *PTH1R* gene expression, TGF-β3-induced production of aggrecan, types I+II collagen) of cartilage FPCs are differential advantages supporting their application in tissue engineering (Broguiere et al., 2016; Studer et al., 2017). Despite expressing stem cell surface markers, cartilage FPCs present relatively lower adipogenic and osteogenic differentiation capacities on a site-specific basis (Stokes et al., 2002; Quintin et al., 2010). Conjugation with alginate optimally stimulates and maintains ECM production, while resisting mineralization and circulatory vessel infiltration *in vivo*, thereby drastically improving stability and therapeutic potential of cartilage FPCs, along with optimal structural parameters (Häuselmann et al., 1994; Mellor et al., 2014; Mhanna et al., 2014; Studer et al., 2017). Polyethylene glycol, chitosan, albumin, or hyaluronan scaffolds have been investigated as functional cell vectors for injectable applications, yielding adhesive, chondrogenic, and mitogenic properties (Madeira et al., 2015; Mardones et al., 2015). For combination product assembly, impermeable, tortuous, and hydrophobic scaffolds often present resistance to liquid phase infiltration, despite high porosity and relative void volume, which negatively affect cell integration, colonization, and persistence (Wendt et al., 2003; Solchaga et al., 2006; Melchels et al., 2010). Various dynamic cell seeding protocols for the induction of active infiltration (e.g., perfusion, centrifugation, orbital shaking, spinner flasks) allow cell distribution uniformity and optimal preservation of cellular integrity and function (Burg et al., 2000; Alvarez-Barreto et al., 2007; Roh et al., 2007; Thevenot et al., 2008). An equilibrium must be reached between cell proliferation and adequate chondrogenesis (i.e., responsiveness versus stability) following homogeneous scaffold seeding, directly defining adequate seeding density, methods for construct obtention, and preculture conditions (Roche et al., 2001; Moretti et al., 2005; Hasegawa et al., 2010; Erickson et al., 2012; Nasrollahzadeh et al., 2017; Studer et al., 2017). This ultimately results in the integration of structural and mass transport properties with the functional chondrogenesis components of the cells, which enable load bearing after successful implantation and integration (Hollister, 2005; Kemppainen and Hollister, 2010). External or internal biochemical modulation, specific processing (e.g., microgel encapsulation), and scaffold mechanical stimulation differentially constitute potent cues for chondrogenesis and structural or functional improvement in bioengineered constructs (Huang et al., 2005, 2010; Campbell et al., 2006; Terraciano et al., 2007; Levinson et al., 2019; Li et al., 2020). Scaffold stiffness improves with ECM deposition and may approach physiological ranges in clinically relevant timeframes (Broguiere et al., 2016). Controlled and function-oriented energy dissipation modulation within native viscoelastic cartilage-like materials favors optimal chondrogenic expression under dynamic loading and subsequent load-bearing (Hunter et al., 2004; Shaw and MacKnight, 2005; Li et al., 2010; Abdel-Sayed et al., 2014). Relatively high energy dissipation levels lead to the upregulation of specific chondrogenic markers (e.g., mRNA of Acan, Col2a1, Sox9, and TGF-β3), while lower dissipation is linked to downregulation (Mauck et al., 2007; Thorpe et al., 2008; Abdel-Sayed et al., 2014).

### Human Tendon FPCs (e.g., FE002-Ten Cell Type) for Regenerative Medicine

Tendinous tissue disorders (e.g., tendinosis, lipoid degeneration, and calcification), along with imperfect inherent tissue healing capacities and iatrogenesis, result in disability, chronic pain, functional, and productivity deficits, particularly in sporting and manual labor areas. These diseases or injuries implicate highly specialized professional care and high burdens for public healthcare systems (Verdan, 1972; Kannus and Józsa, 1991; Maffulli et al., 2003; Sharma and Maffulli, 2005; Tuncali et al., 2005; Reinking, 2012). Adhesions and high rates of secondary ruptures are current clinical concerns, as functionally defective fibrotic scar tissue accumulates (James et al., 2008). Slow inherent tissue metabolism, delayed inflammation, effector recruitment, ECM deposition, tissue architectural reorganization, and alignment render the modulation of tendon regeneration complex (Sharma and Maffulli, 2005; Voleti et al., 2012). The efficacy of tendon transfer is hindered by accelerated graft degeneration and would largely benefit from therapeutic cell stimulation, ideally leading to optimal elasticity, mobility, and tensile strength restoration (O’Brien, 1997; Kannus, 2000). Bioengineering scaffolds of interest, such as human cadaveric and equine decellularized tendons or artificial equivalents, enable optimal maintenance of biocompatibility, mechanical properties, and susceptibility for cell seeding, whereas autologous vestigial tendons remain as the standard of care (Wehbé, 1992; Chong et al., 2009; Jakubietz et al., 2011; Pridgen et al., 2011; Burk et al., 2016; Lovati et al., 2016; Valentin et al., 2016; Aeberhard et al., 2019). Vast arrays of potential therapeutic cell types have been investigated in tendon bioengineering for regeneration enhancement, including tendon sheath fibroblasts, adult tenocytes, stem cells, placenta cells, amniotic cells, and platelet-derivatives (Kadner et al., 2002; Kaviani et al., 2002, 2003; Awad et al., 2003; Chen et al., 2009; Akhundov et al., 2012; Xu et al., 2013; Petrou et al., 2014). Tendon FPCs present tremendous therapeutic potential due to high stability of their tenogenic and karyotypic properties in culture, low propensity for de-differentiation, expansion characteristics, therapeutic stimulatory potential, and the ability to maintain cell viability along with rheological properties of bioengineered hydrogel constructs (Grognuz et al., 2019). Their similarities with stem cells but lack of specific tendon markers require *in vitro* characterization of tendon FPCs using marker panels (e.g., type I collagen, scleraxis, and tenomodulin) (Hulmes, 2002; Le Blanc et al., 2003; Docheva et al., 2005; Murchison et al., 2007; Banos et al., 2008; Taylor et al., 2009). Extensive tendon FPC cell banks may be consistently established and yield approximately 2 × 10^14^ cells within the clinically relevant *in vitro* lifespan, potentially serving for the manufacture of more than 10^8^ treatment units (e.g., reseeded biocompatible scaffolds for localized tendon replacement) (Grognuz et al., 2016b). Relatively increased ECM production is achieved by tendon FPCs under appropriate conditions, as compared to primary adult tenocytes. Development of injectable products designed for tissue regeneration stimulation (e.g., degenerative diseases, small hand injuries, fissures or partial ruptures) using registered medical devices without cell preculture periods enables tangible translational development (Petrou et al., 2014; Grognuz et al., 2016a).

### Human Muscle FPCs (e.g., FE002-Mu Cell Type) for Regenerative Medicine

Intrinsic potential for functional rearrangement and healing is low in human muscle tissue, further diminishing with the advancement of biological age (Grasman et al., 2015; Passipieri and Christ, 2016). Without effective therapeutic management, severe and extensive tissue structural bias (e.g., volumetric muscle loss) is often predictive of poor clinical outcome, as spontaneous optimal healing is hindered or negated, which results in diminished contractility associated with fibrotic tissue formation (Montarras et al., 2005; Ciciliot and Schiaffino, 2010; Grogan et al., 2011; Sicari et al., 2014; Duffy et al., 2016). Muscular tissue engineering is designed to effectively manage and restore structure and function in the aftermath of intense soft tissue trauma, burns, malformations, or tumor ablation, while minimizing volumetric loss and donor-site morbidity consequences (Laurent et al., 2020c). Traditional reconstructive surgical care may tangibly and synergistically benefit from supplementation with cell therapies. Immune rejection, poor distribution, and extremely restricted cell persistence after implantation have been significant challenges limiting the potential of myoblast transfer therapy in muscular loss, Duchenne muscular dystrophy, or cardiac surgery (Partridge et al., 1978; Mendell et al., 1995; Miller et al., 1997; Skuk and Tremblay, 2000; Smythe et al., 2000; Huard et al., 2002; Menasché, 2005). Such obstacles dramatically hamper therapeutic efficacy, as eventual functional benefits are dependent on cell survival *in situ* (Fan et al., 1996; Beauchamp et al., 1997, 1999; Qu et al., 1998; Hodgetts et al., 2000, 2003; Tambara et al., 2003; Sammels et al., 2004). Multimodal development efforts have been allocated to optimize persistence and therapeutic effects of implanted cells, comprising differential cell source choice, cell population purification and pre-treatment, or modulation of existing pharmacotherapeutic care protocols (Huard et al., 1994; Pavlath et al., 1994; Guérette et al., 1997; Qu et al., 1998; Jankowski et al., 2001; Maurel et al., 2005; Schäfer et al., 2006). Defined cell population identity and high purity of human muscle FPCs (i.e., stable desmin expression) or *in vivo* persistence were demonstrated in immunocompetent murine models, excluding immunogenicity and tumorigenicity, while positively affecting contractile recovery potential (Hirt-Burri et al., 2008a; Laurent et al., 2020c). Specific estimations indicate that a single fetal organ donation can potentially yield more than 10^12^ progeny cells at a low passage (i.e., P4), enabling subsequent safe industrial-scale manufacturing of off-the-freezer therapeutic cellular products. High FPC robustness and adaptability to bioengineered scaffolds, such as equine collagen sheets, were shown, with rapid colonization and proliferation of therapeutic cells *in vitro*, and persistence thereof *in vivo* (Hirt-Burri et al., 2008a). Optimal restoration of muscle tissue function was demonstrated, concerning functional endpoints of tissue repair, following engraftment of human muscle FPCs in a murine model for volumetric muscle loss (Laurent et al., 2020c).

### Human Bone FPCs (e.g., FE002-Bone Cell Type)

#### Bone FPCs for Skeletal Tissue Engineering

Conventional specific surgical management strategies for bone injuries or diseases include autografting, allografting, or xenografting, which retain relatively elevated risks of contamination and immune response eliciting, leading to subsequent invasive procedures (Younger and Chapman, 1989; Strong et al., 1996; Vacanti et al., 2001; Schantz et al., 2002; Tenorio et al., 2011). Bone replacement and skeletal regenerative cell therapies focus mainly on orthopedic medicine, osteogenesis imperfecta, and mandibular care (Horwitz et al., 1999; Ohgushi and Caplan, 1999; Yildirim et al., 2000; Bianco et al., 2001; Patino et al., 2002; Rose and Oreffo, 2002; Mauney et al., 2005; Oreffo et al., 2005; Yoshioka et al., 2007; Mendes et al., 2008). Use of FPCs for skeletal tissue engineering eliminates the need for extensive population selection and complex biochemical phenotype manipulation, while cells maintain sustained differentiation states, with relevant mineralization activities *in vitro* and *in vivo* (Petite et al., 2000; Parikh, 2002; Gronthos et al., 2003; Mendes et al., 2004; Montjovent et al., 2004, 2008, 2009). Allogenic FPC supplementation in artificial bone constructs facilitates cell migration, proliferation, and differentiation at the injury site after implantation, in order to favor tissue regeneration (Caplan and Goldberg, 1999; Shea et al., 2000).

#### Bone FPC Modulation and Drug Delivery

Osteogenic activity (e.g., dexamethasone-induced cbfa-1, ALP, type I collagen, and osteocalcin gene expression) and mineralization processes are comparatively superior in magnitude or more rapid in FPCs than in stem cells and adult osteoblasts, whereas orientation toward mature osteoblast differentiation is relatively simple (Zernik et al., 1990; Franceschi, 1999; Karsenty, 2000; Pioletti et al., 2006). Fetal progenitor cell expansion and migration are culture medium-dependent and sensitive to PDGF-BB, FGF-2, or BMP-2 stimulation (Krattinger et al., 2011). Constitutive expression of TGF, VEGF-A, EDN1, IL-6, and MCP-1 in FPCs was shown, along with characteristic markers (e.g., Stro-1, ALP, CD10, CD44, CD54, β2-microglobulin, HLA-I, CD80) (Montjovent et al., 2009). Fetal progenitor cells present a tendency toward osteogenic differentiation, whereas specific modulation is achieved using ascorbic acid, glycerophosphate, 1α,25-dihydroxyvitamin D3, or dexamethasone, and may be evaluated by monitoring the expression levels of *RUNX2*, *OSX*, or *SOX9* (Aubin, 1998; Gallagher, 2003; Krattinger et al., 2011). Bone FPCs display the characteristics of osteoprecursor cells, relatively more advanced in terms of differentiation than stem cells, and produce relatively superior quantities of ECM, whereas fully-induced differentiation processes result in the appearance of specifically mineralized bone-like nodules (Montjovent et al., 2004; Krattinger et al., 2011). Phenotypic maturation *in vivo* was shown to not carry the immune privileges of therapeutic FPCs in rodent models (Hausherr et al., 2017). Chemical functionalization (e.g., click chemistry, bioorthogonal chemical reactions, covalent binding) of therapeutic cell surfaces allows optimal conjugation with bioengineered scaffolds, while maintaining and optimizing cellular viability, adhesion, persistence, and function (Borcard et al., 2011, 2012; Comas et al., 2012; Krauss Juillerat et al., 2012). Optimal mechanical properties and efficient vascularization capacity of implanted constructs are essential, while biodegradable hydrogels may enable local cell maintenance (Tenorio et al., 2011; Amini et al., 2012). For critical-size bone tissue replacement, cyto- and histo-compatible permanent bone-mimicking substitute materials (e.g., bioceramics) must comprise trans-scaffold micro-structure channels enabling nutrient diffusion and migration (i.e., pore size-dependent osteoconduction) of therapeutic cells, to ensure permanent cellularization and sustained functionality (Triplett and Schow, 1996; Ducheyne and Qiu, 1999; Griffith and Naughton, 2002; Montjovent et al., 2007, 2008; Klenke et al., 2008; Krauss Juillerat et al., 2012). The temporal onset of construct preculture mechanical loading influences and regulates bone architectural properties, whereas early or delayed loading may be beneficial for bone tissue formation within short timeframes (Carter et al., 1989; Huiskes et al., 2000; Roshan-Ghias et al., 2010; Boerckel et al., 2012). Based on *in vivo* experiments, it was established that low predictability characterizes the specific behavior of a given cell type and scaffold conjugate, concerning the intensity and temporal onset of mechanical loading (Hausherr et al., 2018). High cellular resistance to shear stress enables extrusion of cell-laden hydrogels through small-bored needles without compromising cellular viability, whereas HA constitutes a versatile and functional scaffold, allowing relatively enhanced cell migration at the delivery site and ameliorated therapeutic stimulation (Drury and Mooney, 2003; Weinand et al., 2006). Similar valuable characteristics (i.e., absorption, biocompatibility, chemotactic activities, void filling, and migration enhancement) are shared by collagen scaffolds (Patino et al., 2002).

### Human Intervertebral Disc FPCs (e.g., FE002-Disc Cell Type) for Regenerative Medicine

The widespread prevalence of intervertebral disc degeneration mainly contributes to back pain-related surgical management and spine surgeries (Urban and Roberts, 2003; Anderson and Tannoury, 2005; Haefeli et al., 2006). Intervertebral disc tissue is characterized by mediocre intrinsic regenerative potential, further complicating therapeutic management and advancing the onset of degenerative disease. Cell therapy approaches for disc degeneration prevention present considerable potential for replacing autologous nucleus pulposus transplantation (Ganey et al., 2003; Sato et al., 2003; Crevensten et al., 2004; Meisel et al., 2006, 2007; Sakai et al., 2006). After intervertebral FPC isolation and during subsequent characterization, both structure and composition of ECM (e.g., aggrecan, type I and II collagen, sulfated GAGs), spontaneously produced by intervertebral disc FPCs, approach those of adult origin, as observed in alginate bead culture, outlining the full chondrogenic differentiation potential (Häuselmann et al., 1994; Mok et al., 1994; Chiba et al., 1997; Melrose et al., 2000; Quintin et al., 2009). Absence of specific markers enabling population purity assessment prompts, for each new fetal organ donation and derived primary cell type, close evaluation of phenotypic consistency and stability for intervertebral disc FPCs, as they represent mixed populations isolated from whole spine units (Quintin et al., 2009). Therefore, based mainly on the initial dissection and culture initiation methods, some cell types may be unfit for further processing and should be excluded at an early stage, based on characterization results. Interestingly, intervertebral disc FPCs presented relatively lower adipogenic differentiation potential than comparable cartilage FPCs (Quintin et al., 2010). Overall, accumulated data strategically positions intervertebral disc FPCs for further research and development in skeletal tissue regeneration applications.

### Human Lung FPCs (e.g., FE002-Lu Cell Type) for Biotechnological Manufacturing or Regenerative Medicine

Lung FPCs present tremendous potential and vast hindsight for applications in biotechnology, as the vaccine industry has been using such cell types for half a century. The finite human diploid MRC-5 cell type was initially isolated in the 1960s from a male fetal lung (i.e., 14-week gestational age), donated following a pregnancy interruption, and has been used as a substrate for manufacture of chickenpox, hepatitis A, polio, smallpox, and rabies vaccines (Jacobs et al., 1970; Lewis and Tarrant, 1972; Petes et al., 1974). Safety, stability, and quality of substrate cell types are of paramount importance, as defects may be passed down to therapeutic products and eventually endanger patients. Some concerns have emerged following reports that MRC-5 fibroblasts could de-differentiate under specific conditions and exhibit different markers typically found in ESCs or MSCs and neural tissue, or further become osteoblasts (Rieske et al., 2005; Zhang et al., 2014; Wan et al., 2018). Such capabilities tend to indicate a relative instability of the considered cell type, potentially creating problems in modern-day industrial validation. Additionally, aging of the MRC-5 cells, recurrent doubts about the identity of currently marketed MRC-5 cells, and unavailability of these in different geographical regions have led to the establishment of replacement cell types. Modern alternatives were reportedly developed (e.g., Walvax-2 cell type, PRC), with particular attention being paid to the ethnicity of the donor, in order to optimize industrial outputs by exploiting shorter doubling times, improved robustness, or cell viability (Ma et al., 2015). Recently established primary lung FPCs, such as the FE002-Lu cell type, benefit from all the aforementioned technical advantages of FPCs, and may be expanded at full industrial scale within specific multi-tiered cell banking workflows, therefore potentially constituting a tangible candidate for the replacement of the MRC-5 cell type. Optimization of novel and safe cellular substrates shall allow for the optimal replacement of biotechnological intermediates for vaccine production, therefore indirectly contributing to augmenting the quality of therapeutic products, benefiting populations globally. Additionally, primary lung FPCs may present substantial therapeutic utility in treating lung tissue inflammatory diseases. Recent clinical studies (i.e., ClinicalTrials.gov identifiers: NCT04315987, Brazil; NCT04313322, Saudi Arabia; NCT04333368, France; ChiCTR2000029990, PRC) are advancing with the use of multiple therapeutic stem cell sources for managing COVID-19 patients (Zhao, 2020). Similarly, it is hypothesized that lung FPCs may provide enhanced anti-inflammatory and tissue regeneration stimulation, as observed within cutaneous regenerative applications of related dermal FPCs. Meanwhile, the tissue-specific origin and high consistency or stability of such cell types may prove to be the optimal parameters for standardized therapeutic success.

### Single Tissue Donation for Multiple Mammalian FPC Types

#### Ovine FPCs and Cell-Based Cell-Free Topical Preparations

**FIGURE 12:**
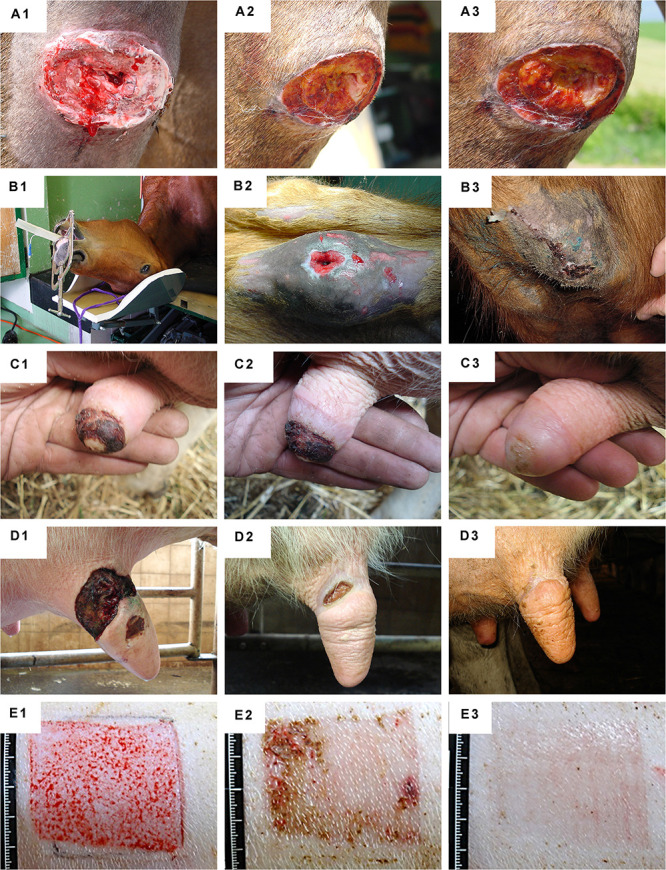
Clinical veterinary case-reports illustrating the use and efficacy of equine Progenitor Biological Bandages, ovine FPC derivatives formulated as creams, and human dermal FPCs formulated in hydrogels for the management of animal traumatic injuries and donor-site wounds. **(A1–A3)** Profound distal limb articular lesion on the right hind knee of a French Saddlebred Pony (i.e., caused by a severe fall against barbed wire). The lesion was treated with ePBBs and bandages were removed after three days. **(B1–B3)** Mandibular fistula created by an abscessed tooth on a Franche-Montagne horse. The lesion was treated with ePBBs and bandages were removed after nine days. **(C1–C3)** Cow udder having suffered compression force trauma. Cell-free cream containing ovine FPC derivatives was applied daily. Photographic representations of the lesions at the beginning of treatment, after two weeks, and after seven weeks. **(D1–D3)** Cow udder having suffered sharp force trauma. The same cream was applied daily. Photographic representations of the lesions at the beginning of treatment, after seven weeks, and after nine weeks. **(E1–E3)** Porcine skin model for donor-site wound healing stimulation evaluation using human dermal FPCs (i.e., FE002-SK2 cell type) formulated in hydrogels. Photographic representations of the lesions at the beginning of treatment, after six days, and after 14 days. Figures modified with permission from Lapp et al. (2013) and Laurent et al. (2020e).

In addition to therapeutic cell roles for tissue-engineered products, banked primary FPCs are well adaptable as intermediates/substrates in the supply chain of therapeutic and medical (e.g., medical devices) or cosmetic/cosmeceutical products, targeting mild to moderate cutaneous diseases or states, such as acne scars, post-laser maintenance, physiological aging marks, burns, and wounds (Limat et al., 1996; Fitzpatrick and Rostan, 2003; Wu et al., 2003; Gold and Biron, 2006; Gold et al., 2007). Various cutaneous/ectodermal and musculoskeletal ovine FPC types (i.e., isolated from skin, muscle, connective tissue) have been established in collaboration with the food industry for further processing, culture-expansion, multi-tiered banking, and the eventual inclusion of cell-free derivatives in stabilized biopharmaceutical topical preparations, achieving further optimization of primary FPC banking for regenerative cutaneous applications (Lapp et al., 2013). Ovine primary FPC types were found to adapt to standardized whole-cell bioprocessing and out-scaling frameworks optimally (i.e., efficiently outperforming human FPC types), characterized by optimal expansion kinetics and remarkable *in vitro* stability (i.e., extensive lifespan, protein concentration regularity), and normalized efficacy in co-culture models. Carefully balanced derivative combinations in near homeopathic relative quantities yielded optimal stimulatory results, indicating complementary or synergistic effects of various specific active principles. Pharmaceutical-grade cell-free preparations were applied for veterinary and human case studies (i.e., wounds and burns), yielding efficient results for aiding tissue repair (Figure 12). Additionally, a significant technological advantage exists in the stabilization of the therapeutic potential of ovine FPCs, consistently retaining and preserving initial physiological properties and therapeutic attributes, *via* derivation of cell-free extracts. In addition, the formulation of the latter in ready-to-use topical pharmaceutical delivery forms with extensive shelf lives compared to fresh living cells is another advantage (Lapp et al., 2013). Such preparations appear well suited for maintenance therapies within consolidated wound repair strategies, or as specific topical regenerative solutions, depending on dosage and formulation type.

#### Equine FPCs in Hippiatric Regenerative Medicine

Based on the extensive experience in the regenerative potential of primary human and ovine FPC types isolated from various cutaneous and musculoskeletal tissues (i.e., skin, muscle, cartilage, tendon, connective tissue, and bone) of single fetal organ donations, the optimized fetal transplantation framework was applied to equine tissue biopsies (i.e., individual organ donations yielding multiple tissue sources) (Laurent et al., 2020b). There is high demand for large animal (e.g., horses or camels) innovative bioengineered therapeutic solutions in tissue reconstruction, which might be extrapolated from human regenerative medicine, due to strong similarities in respective wound healing processes (also see the “One Health Initiative”) (Bigbie et al., 1991; Litzke et al., 2004; Carstanjen et al., 2006; Koch et al., 2009). Primary equine musculoskeletal FPCs were found to optimally and rapidly adapt to standardized bioprocessing and robust multi-tiered biobanking frameworks in view of optimized hippiatric medicine applications (i.e., tissue reconstruction and wound healing). Consistency, safety, and cytocompatibility were demonstrated with collagen and HA constructs, as well as the absence of immunogenicity or tumorigenicity in several case studies of reconstructive surgeries (Laurent et al., 2020b). Indeed, bioengineered equine PBBs (ePBB, formulated as magistral preparations) yielded efficient preliminary results in stimulating healing resurgence and stimulation of animal tissue repair. In particular, equine FPC therapies seemed to effectively stimulate epidermal and soft tissue regeneration, while limiting granulation tissue formation. Allogenic equine FPC therapy products derived from a single equine fetal organ donation may, therefore, be applied for the multifactorial translational musculoskeletal regenerative treatment of millions of veterinary patients in a safe, effective, and cost-effective manner (Laurent et al., 2020b).

## Iterative Optimization of FPC Biobanking and Drug Delivery Options

High clinical and regulatory pressure prompts iterative optimization of bioprocessing methods involving cell culture steps, mainly to replace animal-derived materials or substrates with defined products or consumables compatible with GMP manufacture, for safety and quality maximization (e.g., avoiding risk of contamination by transmissible spongiform encephalopathies in animal serum) (De Corte et al., 2012). Products such as Accutase^®^ or TrypLE^TM^ and Biofreeze^®^, human serum albumin or sugar-based solutions have been proposed to replace porcine trypsin and DMSO-based cryopreservation media, respectively, and numerous producers tentatively develop serum-free culture media or HPL-based supplements, with variable results. The imperative nature of such changes must be relativized, as extensive industrial use of FBS has not yet yielded critical adverse effects. For industrial-scale manufacturing of cell bank lots, stringent optimization must be conducted regarding raw materials, reagents, and contact-process consumable selection. In particular, the make, model, and lot identity of culture vessels and nutrient supplements must be optimized by thorough benchmarking before use in a GMP environment, as these elements may bear significant impacts on endpoint cell yields or population doubling times, thereby tangibly affecting the overall cost of production (Laurent et al., 2020e). Novel culture vessel systems may be investigated for two-dimensional (e.g., Corning^®^ HYPERFlasks^®^, Nunc^TM^ Cell Factories^TM^, or Greiner CELLdiscs^TM^) or three-dimensional (e.g., Terumo Quantum^®^, roller bottles, spinner flasks) cell culture efficiency, but should be thoroughly validated before adoption at industrial scales. Extensive optimization of polymeric biomaterials and novel biophysical processing methods continuously provide delivery scaffold options (i.e., inert, functionalized, or bioactive) for drug delivery of conjugated therapeutic cells. Acceptable cell survival and relative engraftment *in vivo* may be desired in specific applications, as wound environments adversely affect these parameters, due to anoikis, hypoxia, and local inflammatory effectors (Hyun et al., 2013). High resistance to oxidative stress, cryogenic shock, and physical constraints, such as shear stress, enable the effective coupling of primary FPCs with vast arrays of biomaterials, whereas, concerning cytocompatibility, the choice of therapeutic cell type often proves to be a major limiting factor (Ng et al., 2004; De Buys Roessingh et al., 2006; Grognuz et al., 2016a). Importantly, future efforts in the development of therapeutic biological constructs will need to include ancillary, yet necessary, modalities of tissue reconstruction, such as anti-microbial factors to combat infectious complications (Abdel-Sayed et al., 2016; Valerio et al., 2016). In order to pursue continued product optimization and abolition of logistical dependency to cold chains, further biochemical or physical processing of therapeutic cellular materials may be implemented for integral cells or cell-free derivatives, in order to obtain preparations such as desiccated powders (e.g., lyophilizates) or semi-solid topical or injectable formulation (e.g., viscous hydrogels or creams) (Hirt-Burri et al., 2011; Lapp et al., 2013; Aldag et al., 2016; Grognuz et al., 2016a; Bari et al., 2019). For facilitated regulatory submissions, the combination of therapeutic cells should be considered with existing and marketed products, such as medical devices, benefiting from clinical validation. Such approaches, whenever possible, contribute to diminishing the validation efforts of novel TEPs and combination products such as combined advanced therapy medicinal products (cATMPs) in particular (Tenorio et al., 2011). Cell–scaffold interactions, creation of functional tissues, or absence of cytotoxicity and toxic by-products, must then be demonstrated, as well as biodegradability in specific cases (Hirt-Burri and Applegate, 2013; Jafari et al., 2017). Safety risks (i.e., immunogenicity or tumorigenicity) associated with the use of viable therapeutic cells (i.e., stem cells or FPCs) may be completely averted or mitigated by using devitalized cells or cell-free products (e.g., cell-based cell-free formulations). Alternative processing options for cell populations during cellular therapy or product manufacture comprise various physical (e.g., direct cryopreservation, lyophilization) or chemical processes (e.g., controlled lysis), resulting in devitalization and/or loss of cellular structural integrity, which may be followed by extraction or purification steps. Alternatively, conditioned media may be used for cell-free approaches. Devitalization or use of cell-free derivatives may be of considerable interest from a regulatory standpoint, as resulting therapies or products may be classified as cosmetics or medical devices, based on the nature of the intended effects and the relative importance of said effects (i.e., main or ancillary effects). Overall, tangible benefits favor the specific workflows of simultaneous and differential isolation of multiple FPC types from single fetal tissue donations, as culture initiation conditions are highly similar for all considered biopsies, may be controlled in parallel, and may be adjusted iteratively. Further optimization of FPC biobanking shall focus on epigenetics and influences of ethnic diversity on comparative efficiency for therapeutic product design or biotechnological manufacturing optimization.

## Ethics, Morals, Religion, and Politics Around FPCs

The unique approach of multiple-organ harvest following fetal organ donations, as practiced for adult solid organ transplantation, additionally restricts the need for multiple cell type isolation procedures. Technical or logistical availability of fetal tissue is theoretically not an issue, given the high relative rates of voluntary pregnancy termination in modern societies (e.g., six to nine terminations *per* 1,000 women in Switzerland over the past two decades) (Addor et al., 2003; Swiss Federal Statistical Office, 2019). In many countries including Switzerland, procurement of fetal tissue is classified as an organ donation and is highly regulated, as it requires Federal Authorities and Ethics Committee approvals (Applegate et al., 2009, 2010). Furthermore, regulated methodological aspects of Fetal Transplantation Programs and donor consent obtention ensure that related biomedical research does not increase either the number of pregnancy terminations nor the moral value thereof, and does not influence the termination date within the gestational period. Ethical examinations of programs seeking access to such fetal tissues embody a large place in the actual proceedings, whereas large variability exists in this respect throughout different countries and even between different states in Switzerland. Much like work with embryonic cells, the use of FPCs is deeply linked to moral questions, which are most prone to elicit debate. Depending on the technical availability of donated tissues, it is clear that some countries may not establish sufficient amounts of therapeutic treatments, but therapies or cell-based treatments may be imported, where they are allowed for use. In our own personal views, the whole-cell bioprocessing of fetal tissues for the establishment of primary FPC types, as described herein, might appear “unnatural” from conservative or strong religious standpoints. Nevertheless, this workflow requires minimal manipulation and ensures optimal conservation of initial tissue-specific biological characteristics for progeny cell populations. Such cell types therefore require relatively less human and biochemical intervention than phenotypically oriented MSCs or manipulated iPSCs, in order to obtain therapeutic cell populations or cell substrates fit for eventual clinical use.

It is also our personal belief that scientific and ethical advantages may be established around the use of fetal tissue or FPCs for therapeutic purposes, such as the potential medical benefits for millions of patients following one single organ donation, the restricted need for resorting to autograft harvest, and the respectful revalorization of high therapeutic value tissues otherwise destined for destruction. Considered clinical applications and therapeutic benefits resulting from the use of fetal organ donations may be quantified, and their weight may be clearly examined by appropriate regulatory and ethics bodies. Without the original fetal or embryonic cell lines established in the 20th century, many vaccines would not have been developed, potentially costing millions of lives. However, similarly to research on embryonic cells and despite the clear technical and clinical benefits from a scientific point of view, profound ethical and emotional aspects indirectly govern the practice of FPC therapy and the use of such substrates in the biotechnological industry (Lawler, 1981; Sanders et al., 1993; Rahman et al., 1998; Jost, 2002; Zimmerman, 2004; Greely, 2006; Panikkar et al., 2012). In particular, remarkable dissertations by the Catholic Church and Vatican-related groups extensively discuss the use of fetal cells from pregnancy terminations and applied for vaccine production, listing the “incriminated” products and companies and deeming the use of such materials as nuanced between and within the scope of “licit and illicit cooperation in Evil,” under the influence of pharmaceutical companies and pertained to “social/medical moral coercion” (Furton, 1999; Maher et al., 2002; Pontifical Academy for Life, 2006). Interestingly, such positions are not maintained around the use of perinatal stem cells, as their exploitation for therapeutic purposes benefits from more leniency (Abbaspanah et al., 2018; Gaggi et al., 2019). On a political side, direct modulation of fetal cell research (i.e., including *in vitro* fertilization) has been achieved in the United States by cyclic restrictions on federal funding, with conservative positions aiming at banning such practices, while liberals have historically promoted women’s health and freedom of choice, directly and indirectly benefiting medical progress (Manier, 2002). In a broader perspective, it is to note that perceived obstructionism to specific therapeutic cell source exploitation is not limited to religious or radical positions, as the US government has, through various and evolving polices, banned many aspects of research around ESCs for example, as this specific topic remains in heated debate (Murugan, 2009).

## Legal and Regulatory Frameworks for FPCs and Product Development

Development and commercialization of therapies or cell-derived products are highly regulated in order to ensure safety and quality for the recipient. Respective regulatory landscapes and frameworks have been disruptively updated in Europe recently, creating labyrinthian procedures with mitigated outcomes on advances in the field of regenerative medicine, while potentially creating many regulatory pathway complications and deadlocks (Bertram et al., 2012; De Wilde et al., 2016; Dimitropoulos et al., 2016; Hartmann-Fritsch et al., 2016; Abdel-Sayed et al., 2019b). Bioengineered products (e.g., cell-laden scaffolds), as considered herein for primary FPC delivery, are classified as combined ATMPs or TEPs, implying inherent substantial manipulations for standardized transplant elaboration, for which GMP requirements are derived from classical pharmaceutical industry guidelines (Johnson et al., 2011; Fisher and Mauck, 2013; Esteban-Vives et al., 2018, 2019). Such dangerous or hampering constraints have limited and eventually reduced the number of products and therapies reaching the market in Europe and are particularly problematic for University Hospitals in particular, as local regulators enforce supranational regulatory frameworks often in detrimental or jeopardizing ways concerning historically used and clinically proven therapies (e.g., cultured autografts for burn patients) (Gallico et al., 1984; Gallico and O’Connor, 1985; Hickerson et al., 1994; Wood et al., 2006; Cirodde et al., 2011; Auxenfans et al., 2015; Eder and Wild, 2019; Laurent et al., 2020i). Faced with pharaonic costs of GMP manufacture and regulatory submissions burdening all public and private stakeholders, hospitals have developed differential approaches to implement in-house cell therapies (Gaspar and Swift, 2013; Cuende et al., 2014). Such undertakings were essential in order to comply with overarching legal frameworks, while continually providing the best therapies available to patients and conducting tangible innovative translational research for highly specialized medical applications. Such approaches of legal exposure mitigation comprise hospital exemptions, compassionate use, exceptional authorizations, orphan drug pathways, magistral or officinal preparations, possibly paving the way for the inclusion of cell-based therapies or cell/cell-derived APIs in official recognized repositories such as pharmacopeias (Pirnay et al., 2013, 2018; World Medical Association, 2013; Pearce et al., 2014; Dimitropoulos et al., 2016; Laurent et al., 2020i). Conjugation of high innovation and virtuosic interpretation of restrictively rigid or unharmonized legal and regulatory frameworks are current necessities, in order to ensure the progress of translational therapeutic developments for the benefit of patients worldwide.

## General Discussion

The present work describes fundamental, preclinical, clinical, and industrial developments embodying the scientific advances supported by Swiss FPC banking. Such comprehensive reformulation and update of the past three decades of multidisciplinary work aimed to substantiate and convey interest, broadening awareness and use of standardized protocols for translational regenerative medicine, potentially impacting millions of patients suffering from cutaneous and musculoskeletal wounds and diseases. The high utility potential of recently derived primary FPC types (e.g., FE002-Lu cell type) for biopharmaceutical therapeutic product manufacturing was also addressed, allowing for potential direct, indirect, and synergistic improvement of modern therapeutic armamentariums. The necessity for safe and consistent biological material sources is of paramount importance, in view of applicable regulatory, technical, and economic requirements existing within cell therapy product or biotechnological substrate development. In such regulated and defined contexts, optimization and standardization are of prime concern and should be the key steps in any translational workflows and manufacturing processes. Optimal management of safety and consistency of therapeutic cell sources is accomplished by avoiding pooling of numerous heterogeneous biological samples, and alternatively, exploiting sustainable multi-tiered FPC biobanks, simultaneously and differentially established after single fetal organ donations. Iterative therapeutic optimization and customized Fetal Transplantation Programs, enabling ethical and controlled biological material revalorization, have constituted the core innovative and developmental base for FPC therapy in Switzerland throughout three decades (Figure 1). Straightforward workflows were devised for tissue procurement, maximizing traceability, safety, consistency, and robustness of progeny cellular materials (Figures 6–8). The overall perception generated by translational work on FPC banking and transposition of related innovative biomedical technologies has comprehensively detailed the complexity of technical and therapeutic success obtention, which remains as a founding prerequisite in commercial product development. Banked FPCs have been historically used and thoroughly investigated throughout three decades in Switzerland, and have been deemed to adapt exceptionally well to specific therapeutic product developmental pathways. Extensive clinical experience has demonstrated the safety and usefulness of multiple primary FPC types to date. In the Lausanne University Hospital, pioneer contributions to innovative cutaneous regeneration solutions using dermal FPCs (e.g., FE002-SK2 cell type) have constituted the unified clinical flagship and eventual translational embodiment of the Swiss FPC Transplantation Program (Figures 9–12). These undertakings have yielded plethoric insights into the adequate conjugation of modern biotechnological innovation with current constraining legislative, ethical, and regulatory frameworks.

**FIGURE 13:**
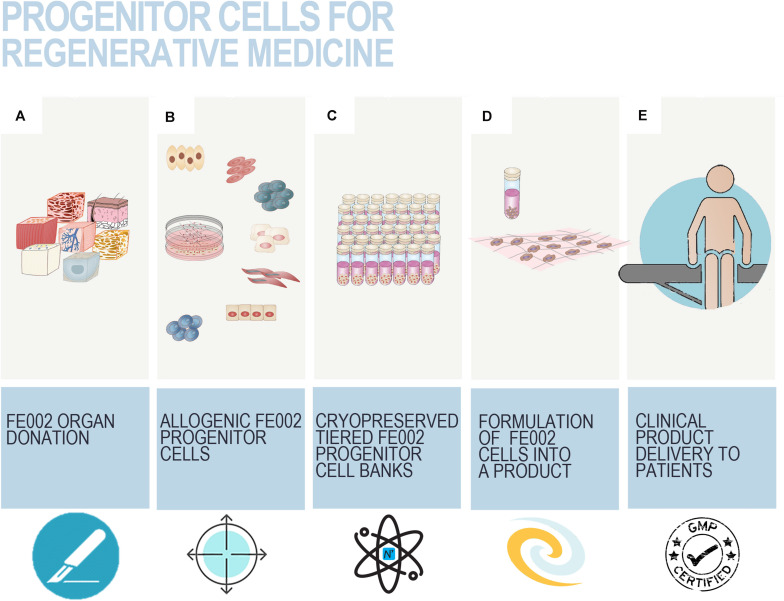
Workflow schematic from initial fetal organ donation biopsy processing to FPC drug delivery to the patient within regenerative medicine settings. Following procurement of the FE002 donation **(A)** within the redefined regulatory framework (i.e., post-2007), tissue-specific allogenic primary FPC types were differentially and simultaneously derived **(B)**, and used to constitute multi-tiered cryopreserved cell banks **(C)**. In view of clinical delivery of therapeutic cells, appropriate vials may be initiated from storage and conjugated with adequate bioengineered scaffolds **(D)**. The resulting constructs are standardized and safety is ensured by GMP processing from raw materials to final products. Following liberation, the products are transferred to the clinic for application on patients **(E)**.

**FIGURE 14:**
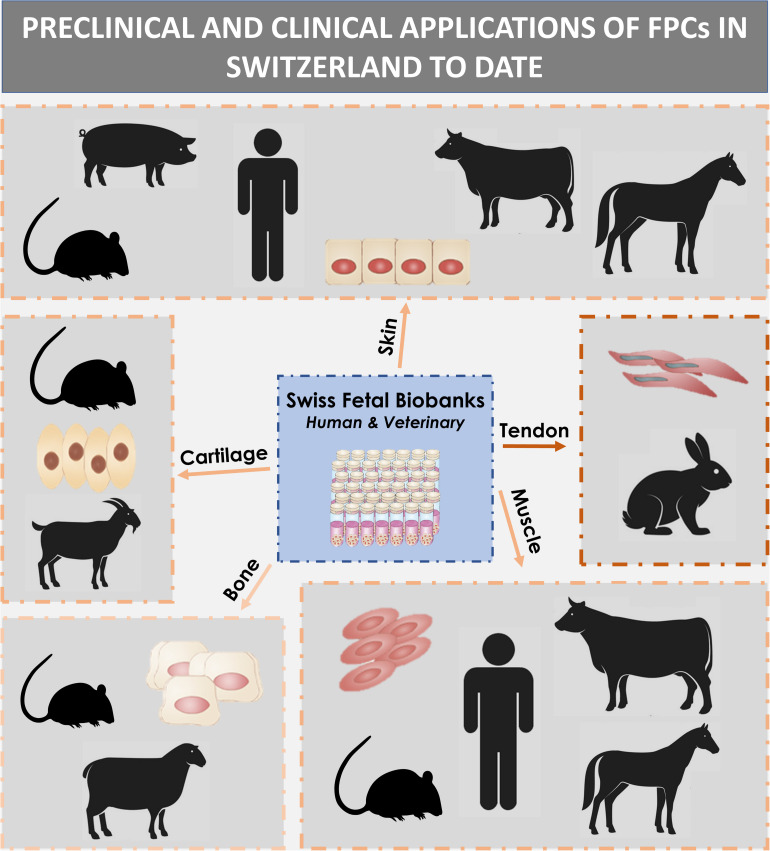
General overview of *in vivo* experimentation and clinical applications of banked primary FPC types in Switzerland during the past two decades. Therapeutic cells constituted successive human Fetal Biobanks, as well as equine and ovine Fetal Biobanks. Therapeutic products comprised combined cell therapy products (i.e., PBBs, ePBBs, viable cells seeded in alternative polymeric scaffolds) or cosmeceutical/medical device-type semi-solid topical formulations of cell-free extracts (i.e., creams and hydrogels). Each FPC type is associated with the different models (i.e., human, porcine, murine, ovine, caprine, bovine, equine, lagomorph) which were part of preclinical investigations or clinically treated with FPCs or stabilized derivatives thereof. Accumulated clinical experience and hindsight attest to the absence of immunogenicity or tumorigenicity of mammalian FPCs, in their bio-integral, viable, or cell-free extract form, in both allogenic and defined xenogenic settings.

Transversal works on soft tissue and musculoskeletal FPC types of human and animal origin have provided diversified and differential insights into the potentials of FPC banking and supported further translational work in clinical testing and implementation. Most importantly, a single human fetal organ donation (i.e., FE002) qualifying for the Swiss Fetal Transplantation Program in 2009 yielded multiple unique FPC types (e.g., skin, cartilage, tendon, muscle, bone, and lung FPCs), validating the sustainable model of single donation for simultaneous differential organ harvest, subsequently presenting the *quasi*-infinite potential of applied research, clinical studies, and product development (Figures 7, 13). Widespread optimized and standardized sustainability constitute the core therapeutic value of FPC material sourcing and biobanking workflows supported herein, allowing the potential derivation of billions of affordable and efficient therapeutic product doses. As demonstrated herein by the comprehensive and detailed holistic approach of Swiss FPC biobanking technology, a single voluntary fetal organ donation is sufficient to support translational research encompassing the cutaneous and musculoskeletal systems for several decades (Figure 14 and Table 1). Further formulation and delivery system optimization, preclinical work, and clinical translation of therapies using FPCs will further enhance quality and efficiency of therapeutic care, benefiting overall health of patients worldwide.

## Data Availability Statement

The raw data supporting the conclusions of this article will be made available by the authors, without undue reservation.

## Ethics Statement

The studies involving human participants were reviewed and approved by the CHUV University Hospital: State Ethics Committee. Written informed consent to participate in this study was provided by the participants’ legal guardian/next of kin. Written informed consent was obtained from the individual(s) for the publication of any potentially identifiable images or data included in this article.

## Author Contributions

AL and LA: study conception and design. AL, CS, MM, and NH-B: acquisition of data. AL and NH-B: analysis and interpretation of data. AL, NH-B, MM, and LA: drafting of the manuscript. CS, NH-B, AB, WR, and LA: critical revision. AL, CS, MM, AD, WR, NH-B, and LA: acceptance of final manuscript. All authors contributed to the article and approved the submitted version.

## Conflict of Interest

AL was employed by companies Tec-Pharma SA and LAM Biotechnologies SA. The remaining authors declare that the research was conducted in the absence of any commercial or financial relationships that could be construed as a potential conflict of interest.

## Abbreviations

API: active pharmaceutical ingredient
ASC: adipose stem cell
ATMP: advanced therapy medicinal product
BMSC: bone marrow stromal cell
BPyV: bovine polyomavirus
cATMP: combined advanced therapy medicinal product
CD: cluster of differentiation
cGMP: current good manufacturing practices
CHUV: centre hospitalier universitaire vaudois
CMV: cytomegalovirus
DMEM: Dulbecco’s modified Eagle medium
DMSO: dimethyl sulfoxide
DNA: deoxyribonucleic acid
EBV: Epstein-Barr virus
ECM: extracellular matrix
EOPCB: end of production cell bank
ePBB: equine progenitor biological bandage
EPC: endothelial progenitor cell
FBS: fetal bovine serum
FPC: fetal progenitor cell
GF: growth factor
GMP: good manufacturing practices
HA: hyaluronic acid
HAV: hepatitis A virus
HBoV: human bocavirus
HBV: hepatitis B virus
hCMV: human cytomegalovirus
HCV: hepatitis C virus
HSC: hematopoietic stem cell
HE: hematoxylin and eosin
HHV-6/7/8, human herpes viruses types 6: 7 and 8
HIV-1/2: human immunodeficiency viruses types 1 and 2
HLA: human leukocyte antigen
HPL: human platelet lysate
HPV: human papillomavirus
HTLV-1/2: human T-cell leukemia-lymphoma viruses types 1 and 2
HuPyV: human polyomavirus
IB: investigator’s brochure
IMPD: investigational medicinal product dossier
iPSC: induced pluripotent stem cell
itMSC: ischemia-tolerant mesenchymal stem cell
KIPyV: KI polyomavirus
LSC: limbal stem cell
MCB: Master Cell Bank
MoA: mechanism of action
mRNA: messenger ribonucleic acid
MSC: mesenchymal stem cell
NSC: neural stem cell
PBB: progenitor biological bandage
PCB: Parental Cell Bank
PCR: polymerase chain reaction
PDT: population doubling time
PDV: population doubling value
PS: penicillin-streptomycin
Px: passage number x
RNA: ribonucleic acid
SV40: simian virus 40
TEM: transmission electron microscopy
TEP: tissue engineering product
WCB: Working Cell Bank
WUPyV: WU polyomavirus.
