# Supplementary material for: Holistic Approach of Swiss Fetal Progenitor Cell Banking: Optimizing Safe and Sustainable Substrates for Regenerative Medicine and Biotechnology
Source: Front Bioeng Biotechnol. 2020 Oct 23;8:557758. doi: 10.3389/fbioe.2020.557758 (PMC7644790; doi:10.3389/fbioe.2020.557758)
Supplement: Supplementary file 1 [file Image_1.pdf]

SUPPLEMENTARY MATERIAL

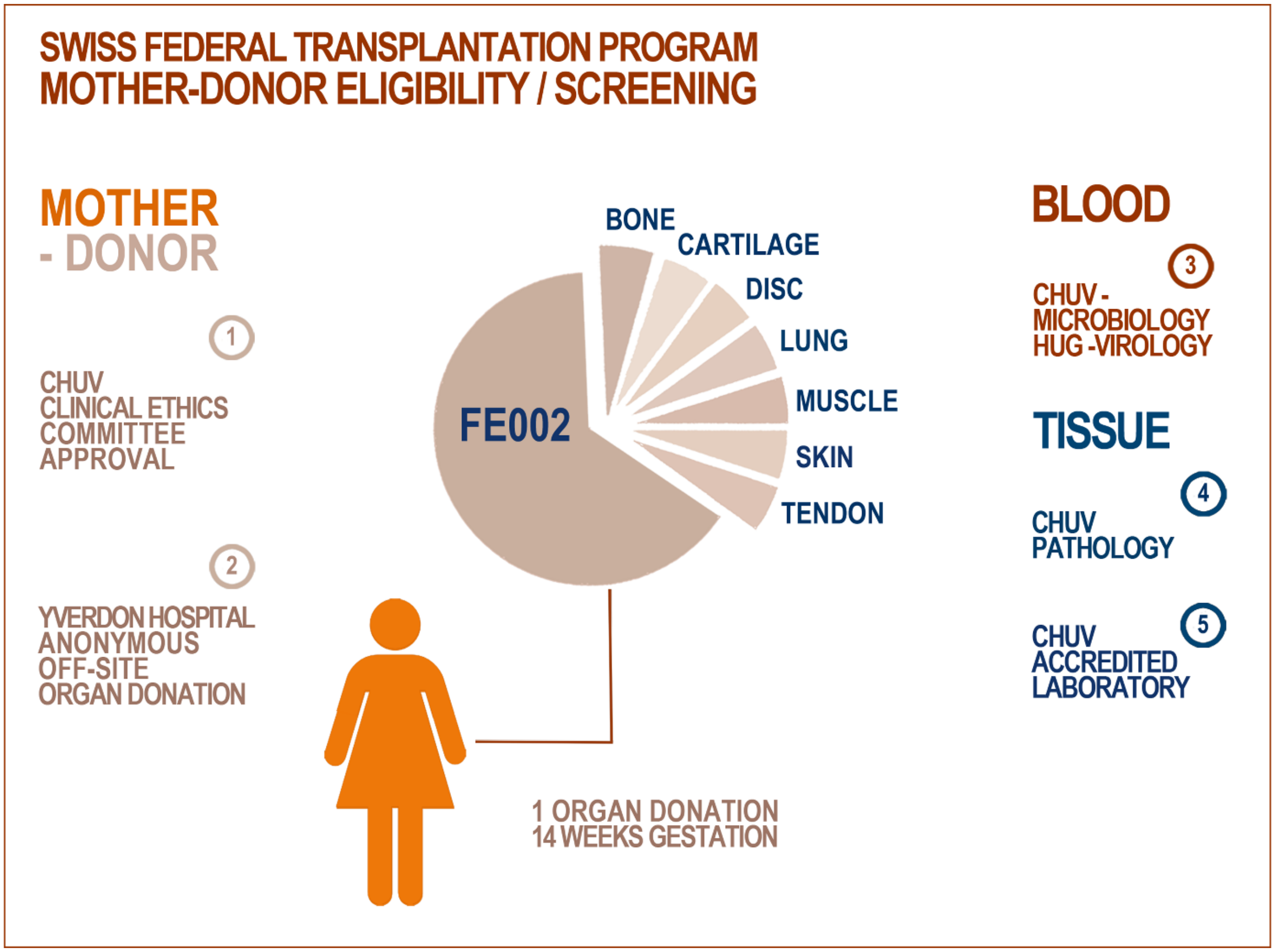

Supplementary Figure S1 | Overview of the Swiss Federal Fetal Transplantation Program practical workflow, as it was devised to adapt to legal and regulatory updates in 2007. This workflow was used in 2009 for the procurement and processing of two fetal organ donations, the latter being the FE002 donation. **(1)** In order to initiate the Transplantation Program, proper approval was obtained from the CHUV University Hospital Medical Ethics Committee, in order to activate the procedures and workflows for establishing fetal biobanks for clinical and research purposes. **(2)** Identification of a qualifying mother-donor providing informed consent was performed in a secondary hospital (i.e., Yverdon-les-Bains, Switzerland), in order to guarantee strict independence between the medical team in charge of the pregnancy termination and the team responsible for fetal biobank establishment and characterization. **(3)** Repeated serology testing of the mother-donor excluded vertical transmission of major adventitious agents (i.e., HBV, HCV, HSV, EBV, HIV-1/2, HTLV-1/2, CMV, S-West Nile virus, *Toxoplasma gondii*, *Treponema pallidum*). Viral testing was performed in the HUG Geneva University Hospital. **(4)** A full pathophysiological examination was carried out for confirmation of physical and physiological normality of the donation. **(5)** Micro-dissection of specific tissues was performed, and samples were procured for bioprocessing and FPC *in vitro* culture initiation. Figure adapted from Laurent *et al.*, 2020e. **CHUV** Centre Hospitalier Universitaire Vaudois; **FPC** Fetal Progenitor Cell; **HUG** Hôpitaux Universitaires de Genève.

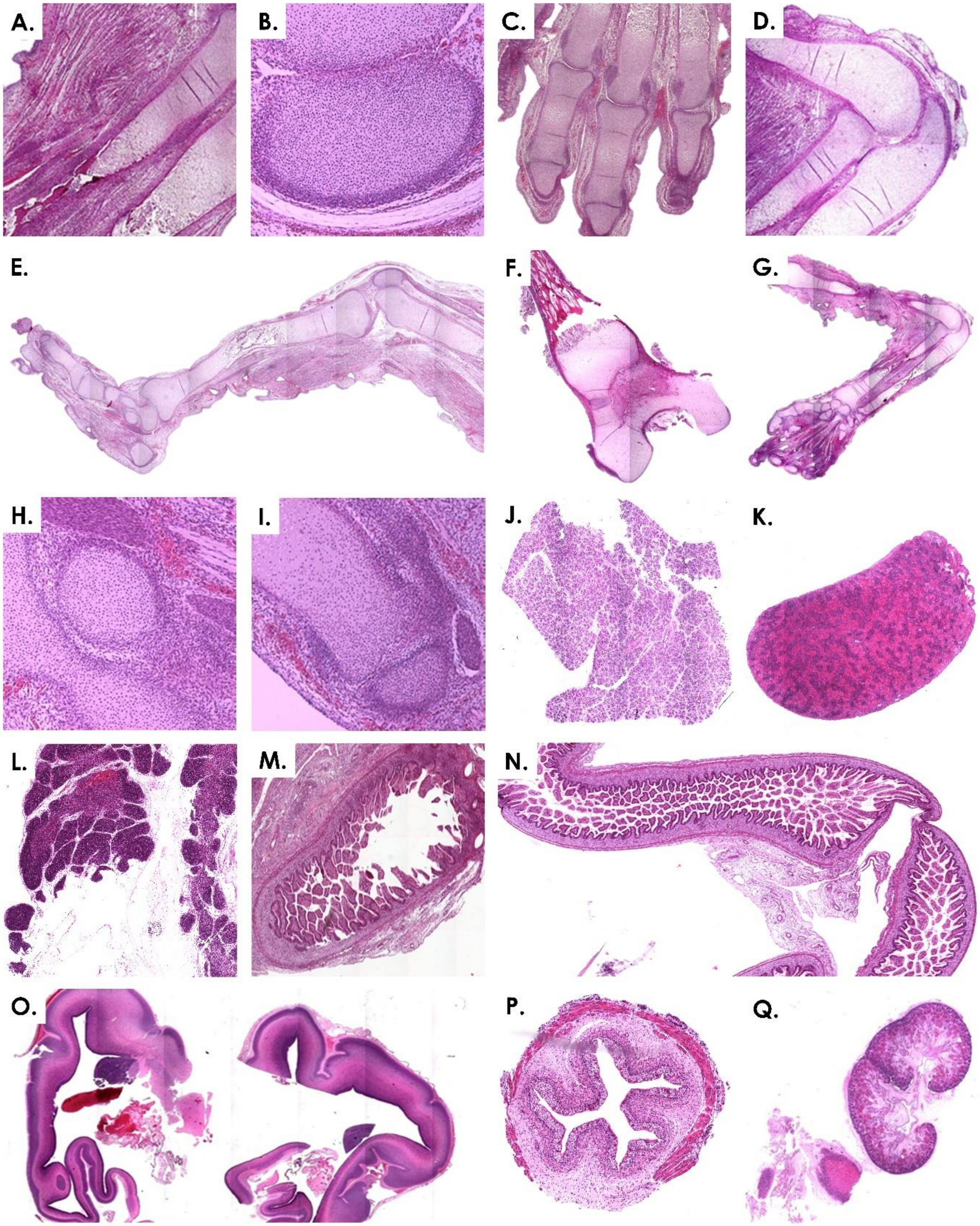

Supplementary Figure S2 | Composite photographic imaging of histopathological investigation of the FE002 organ donation. Tissues were appropriately embedded and sliced before staining with hematoxylin and eosin (HE). **(A)** Internal face of the left elbow. **(B)** Kneecap. **(C)** Fingers from the right hand. **(D)** Overview of the left elbow structure. **(E)** Overview of the right leg. **(F)** Femoral bone head. **(G)** Overview of the left arm. **(H)** Dense tissue from the ankle. **(I)** Toe. **(J)** Lung tissue, right lobe. **(K)** Left gonad. **(L)** Thymus tissue. **(M)** Lumen of the appendix. **(N)** Duodenal extremity. **(O)** Cerebral structures. **(P)** Lumen of the descending colon. **(Q)** Left kidney and suprarenal gland.

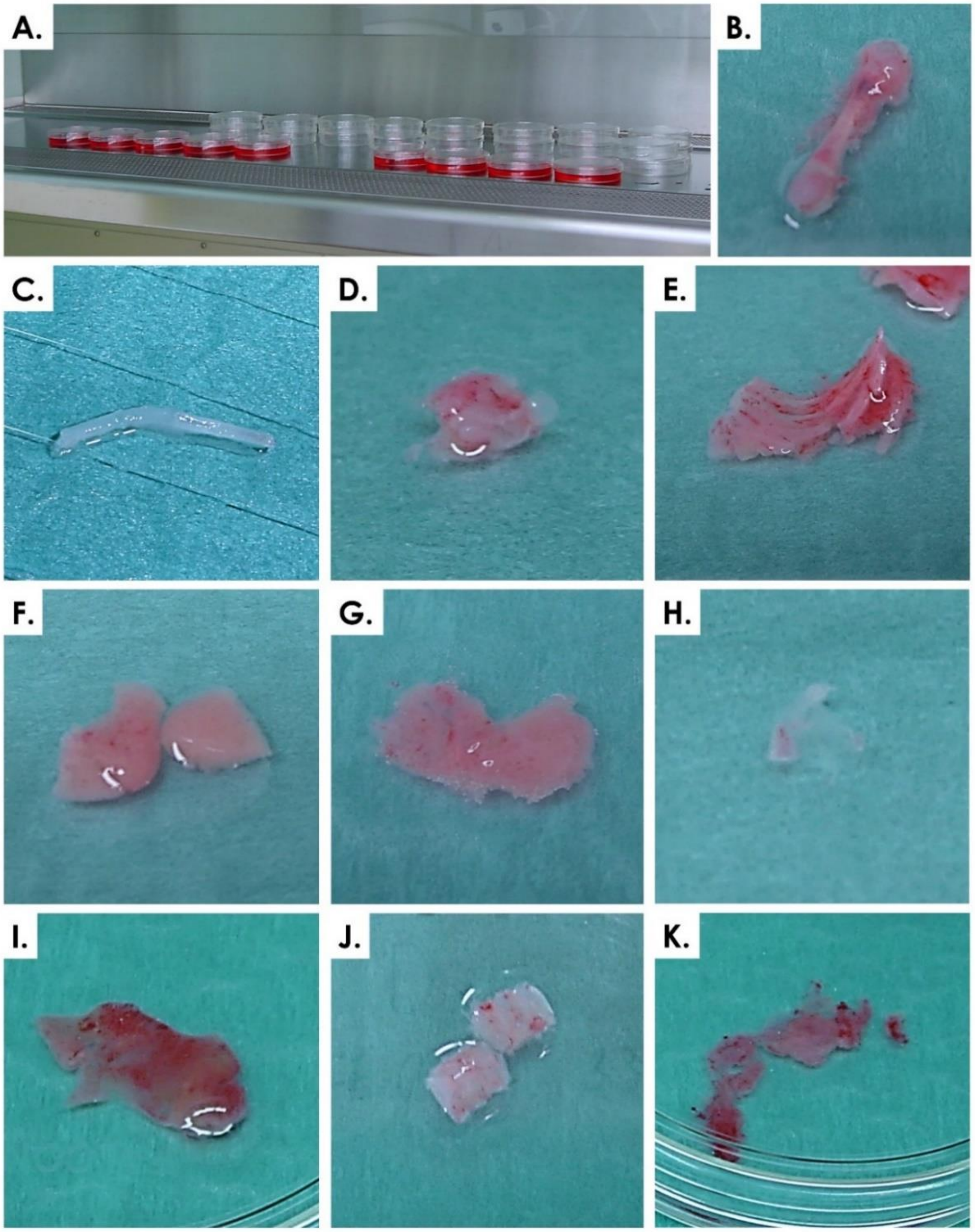

Supplementary Figure S3 | Photographic imaging of simultaneous differential biopsy processing following a fetal organ donation (i.e., FE002, 2009). Representative pictures of the provided specific tissue samples, before differential treatment (i.e., enzymatic versus non-enzymatic) and FPC *in vitro* culture initiation. The whole process was performed and validated twice, with the FE002 donation qualifying for subsequent clinical applications. **(A)** Overview of the laminar flow hood in preparation for enzymatic and non-enzymatic processing of fetal tissue biopsies following procurement (i.e., FE002 donation). **(B)** Fetal bone tissue serving for the establishment of bone FPC types (e.g., FE002-Bone cell type). **(C)** Fetal cartilage tissue serving for the establishment of cartilage FPC types (e.g., FE002-Cart cell type). **(D)** Fetal articular cartilage tissue serving for the establishment of articular cartilage FPC types (e.g., FE002-Cart.Art cell type). **(E)** Fetal connective tissue serving for the establishment of connective tissue FPC types (e.g., FE002-CT cell type). **(F)** Fetal lung tissue serving for the establishment of lung FPC types (e.g., FE002-Lu cell type). **(G)** Fetal muscle tissue serving for the establishment of muscle FPC types (e.g., FE002-Mu cell type). **(H)** Fetal tendon tissue serving for the establishment of tendon FPC types (e.g., FE002-Ten cell type). **(I)** Fetal ventral skin serving for establishment of dermal FPC types (e.g., FE002-SK1 cell type). **(J)** Fetal spine and intervertebral disc tissue serving for the establishment of intervertebral disc FPC types (e.g., FE002-Disc cell type). **(K)** Fetal dorsal skin serving for the establishment of dermal FPC types (e.g., FE002-SK2 cell type) or epidermal FPC types (e.g., FE002-KER cell type).

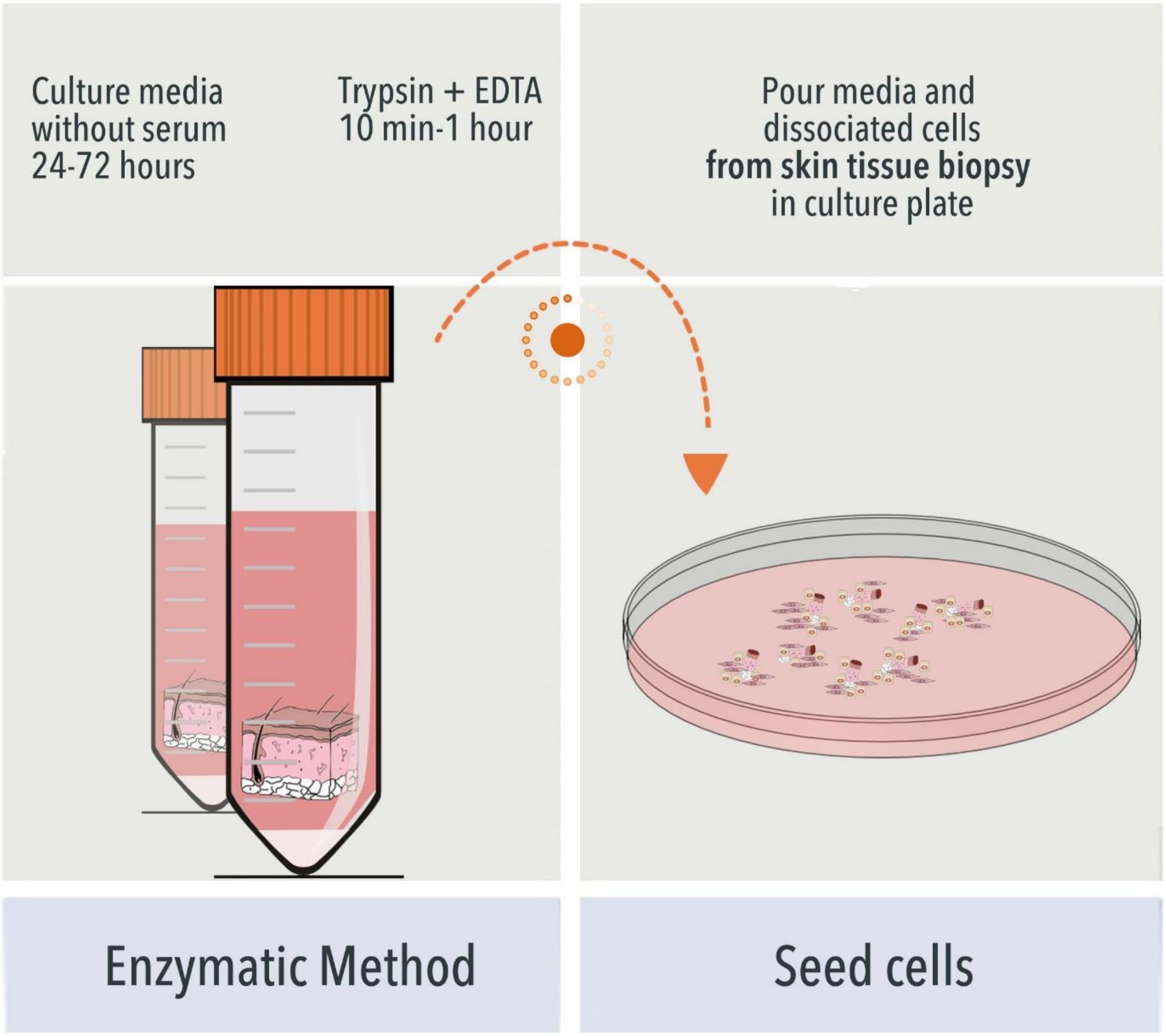

Supplementary Figure S4a | Schematic representation of the enzymatic method of fetal biopsy processing (e.g., fetal skin sample) for adherent FPC *in vitro* culture initiation. For optimal results, the biopsies should be procured by the pathology department after micro-dissection and transferred in a DMEM-based solution (i.e., containing no FBS). The samples should thereafter be stored for 24-72 hours at 4°C. The best results are obtained by further processing the samples after 24 hours of incubation at 4°C, whereas the biopsies are transferred into prepared digestion tubes (i.e., containing trypsin-EDTA) and incubated at 37°C for 10-60 minutes, depending on the sample type and size. Skin and muscle samples are rapidly dissociated for example, while bone or cartilage samples require relatively longer digestion periods. After proper trypsin inactivation by adjunction of FBS, the resulting cell suspension may be rinsed and transferred to culture dishes. To remove the inactivated digestion solution, the suspension may be centrifuged at 230 x *g* for 10 minutes, before the cells are resuspended in appropriate culture medium. Tissue culture dishes are subsequently incubated appropriately to favor adherent FPC culture initiation. **DMEM** Dulbecco's Modified Eagle Medium; **EDTA** Ethylenediaminetetraacetic Acid; **FBS** Fetal Bovine Serum.

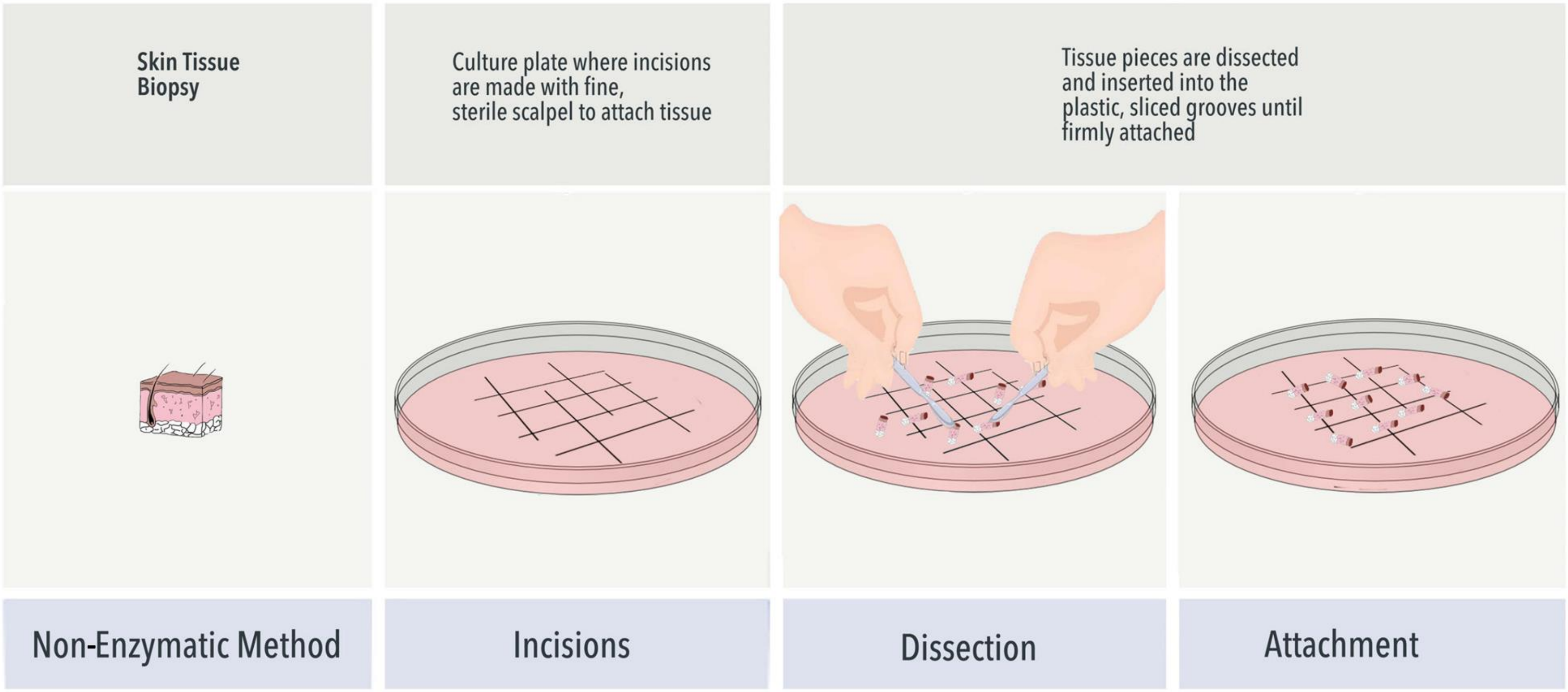

Supplementary Figure S4b | Schematic representation of the non-enzymatic method of fetal biopsy processing (e.g., skin sample) for adherent FPC culture initiation and Parental Cell Bank establishment. This method is described in the related patent “Preparation of Parental Cell Bank from Foetal Tissue”, N°WO 2013/008174 A1, Applegate LAA, 2013. In the same way as for the enzymatic method, optimal results are obtained if the fetal biopsies are pre-conditioned with a 4°C incubation period of at least 24 hours in serum-free medium. The appropriate amounts of non-coated tissue culture plates are thereafter pre-conditioned as well, by means of aseptic and deep scoring of the culture surfaces with scalpels in a checkboard pattern. Secondary biopsy fragments are then transferred to the culture vessels and further minced. Resulting tissue particulates are finally homogenously distributed on the scored culture surface and are attached by insertion in the grooves. Such processing, along with limited initial culture medium volume use, reduces the probability of tissue detachment and flotation, which would compromise adherent FPC culture initiation. Culture dishes are subsequently incubated appropriately to favor adherent cell culture initiation.

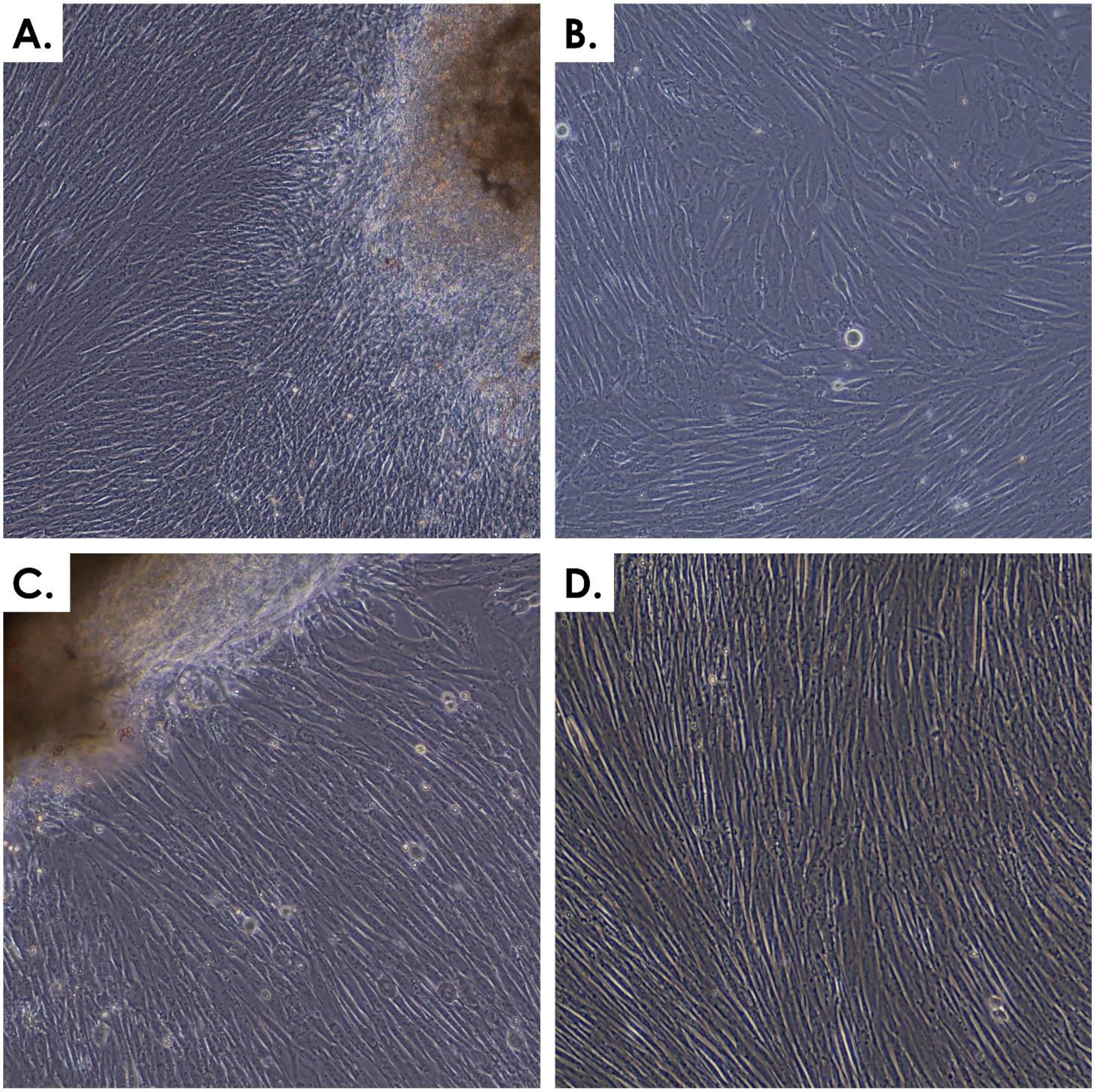

Supplementary Figure S5a | Photographic imaging of FPC *in vitro* culture initiation and culture-expansion steps performed following a single fetal organ donation, within updated legislative frameworks (i.e., post-2007) in Switzerland. Various fetal tissue biopsies were procured from the same organ donation (i.e., donation FE002, 2009) for simultaneous and differential processing, following both enzymatic and non-enzymatic methodologies for *in vitro* cell culture initiation. Pictures were obtained under 200X optical magnification on a phase contrast microscope and represent the non-enzymatically isolated primary FPC types. **(A-B)** Ventral fetal skin fragment with emitting dermal FPCs (i.e., FE002-SK1 cell type, P0). **(C-D)** Dorsal fetal skin fragment with emitting dermal FPCs (i.e., FE002-SK2 cell type, P0) and confluent cells at P2. This cell type was deposited with the accession number ECACC 12070301-FE002-SK2 in July 2012. **ECACC** European Collection of Authenticated Cell Cultures.

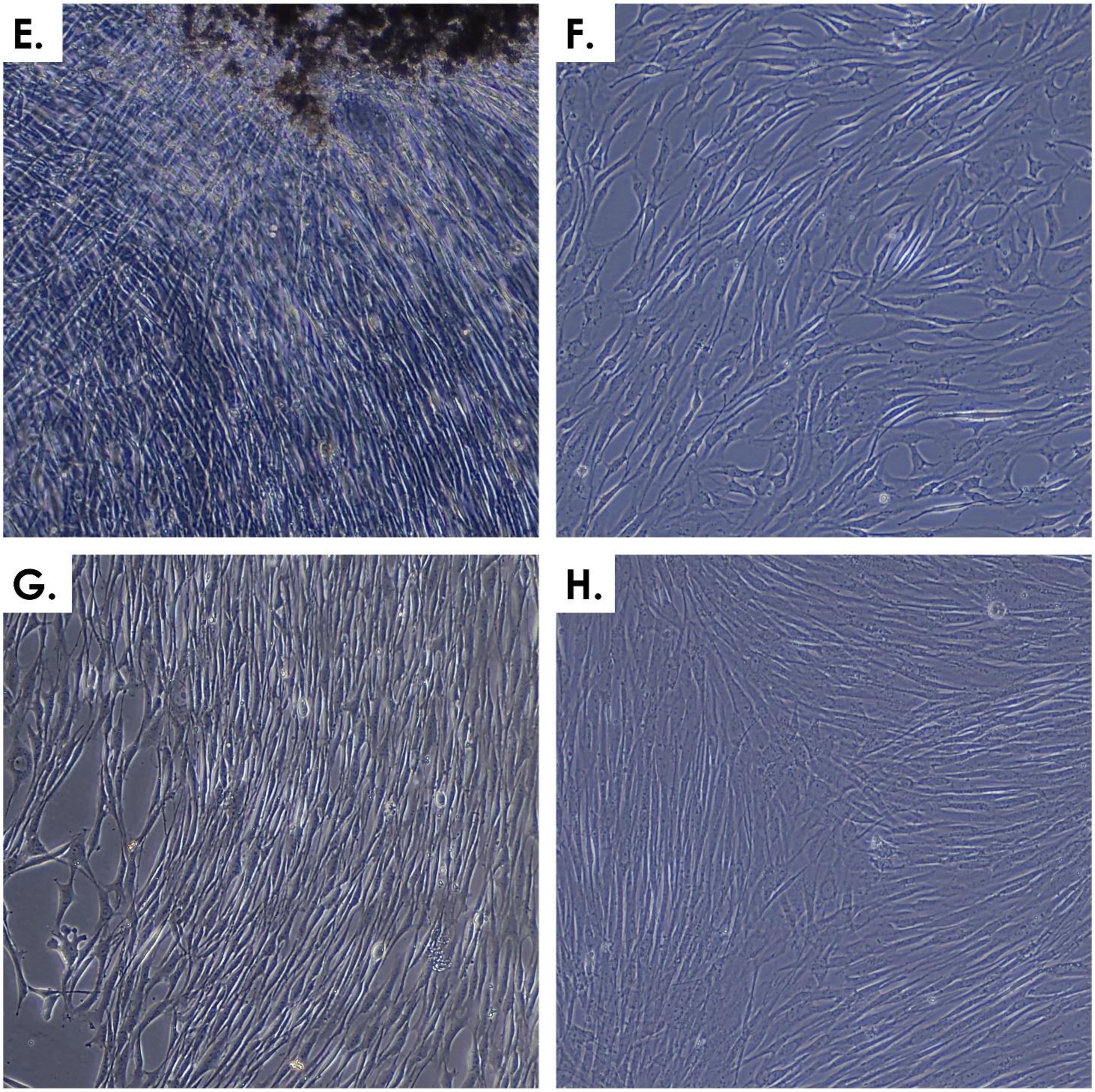

Supplementary Figure S5b | Photographic imaging of FPC *in vitro* culture initiation and culture-expansion steps performed following a single fetal organ donation, within updated legislative frameworks (i.e., post-2007) in Switzerland. Various fetal tissue biopsies were procured from the same organ donation (i.e., donation FE002, 2009) for simultaneous and differential processing, following both enzymatic and non-enzymatic methodologies for *in vitro* cell culture initiation. Pictures were obtained under 200X optical magnification on a phase contrast microscope and represent the non-enzymatically isolated primary FPC types. **(E-F)** Fetal tendon tissue with emitting tendon FPCs (i.e., FE002-Ten cell type, P0). This cell type was deposited with the accession number ECACC 12070302-FE002-Ten in July 2012. **(G-H)** Articular cartilage FPCs (i.e., FE002-Cart.Art cell type, P0) and confluent cells at P2. This cell type was deposited with the accession number ECACC 12070303-FE002-Cart in July 2012. **ECACC** European Collection of Authenticated Cell Cultures.

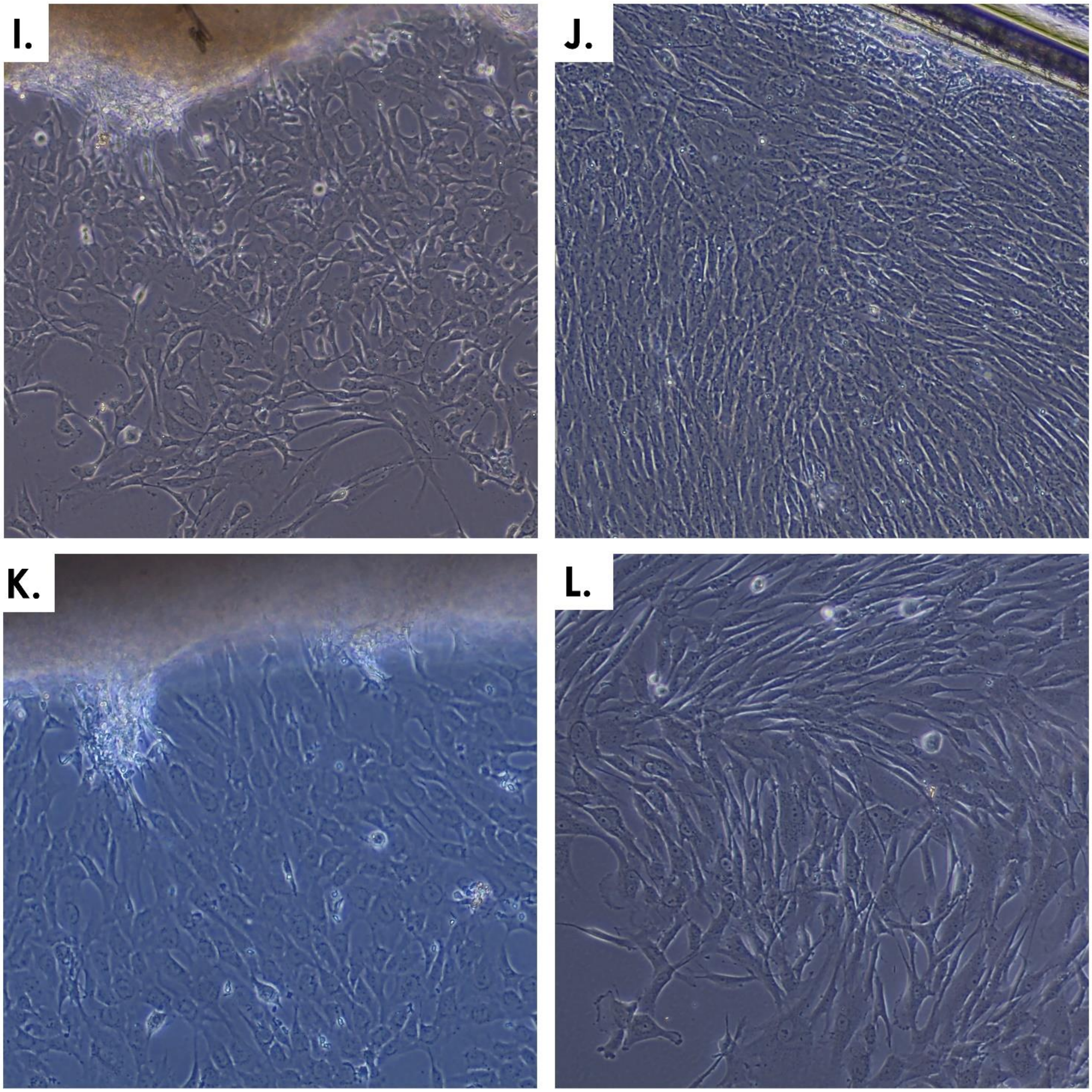

Supplementary Figure S5c | Photographic imaging of FPC *in vitro* culture initiation and culture-expansion steps performed following a single fetal organ donation, within updated legislative frameworks (i.e., post-2007) in Switzerland. Various fetal tissue biopsies were procured from the same organ donation (i.e., donation FE002, 2009) for simultaneous and differential processing, following both enzymatic and non-enzymatic methodologies for *in vitro* cell culture initiation. Pictures were obtained under 200X optical magnification on a phase contrast microscope and represent the non-enzymatically isolated primary FPC types. **(I-J)** Fetal cartilage tissue with emitting cartilage FPCs (i.e., FE002-Cart cell type, P0). **(K-L)** Fetal bone tissue with emitting bone FPCs (i.e., FE002-Bone cell type, P0).

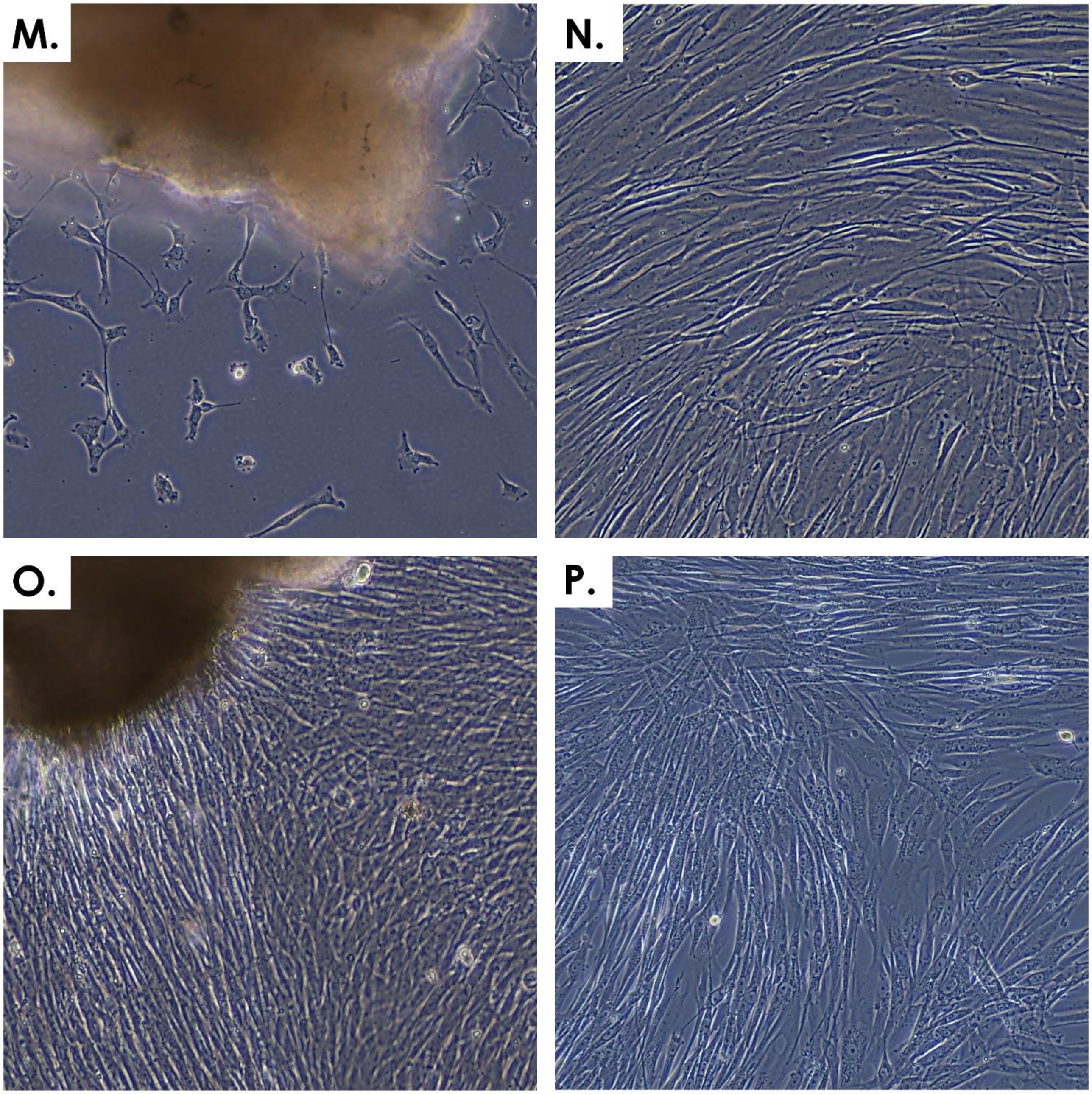

Supplementary Figure S5d | Photographic imaging of FPC *in vitro* culture initiation and culture-expansion steps performed following a single fetal organ donation, within updated legislative frameworks (i.e., post-2007) in Switzerland. Various fetal tissue biopsies were procured from the same organ donation (i.e., donation FE002, 2009) for simultaneous and differential processing, following both enzymatic and non-enzymatic methodologies for *in vitro* cell culture initiation. Pictures were obtained under 200X optical magnification on a phase contrast microscope and represent the non-enzymatically isolated primary FPC types. **(M-N)** Fetal intervertebral disc tissue with emitting disc FPCs (i.e., FE002-Disc cell type, P0) and confluent cells at P1. **(O-P)** Fetal lung tissue with emitting lung FPCs (i.e., FE002-Lu cell type, P0) and confluent cells at P1.

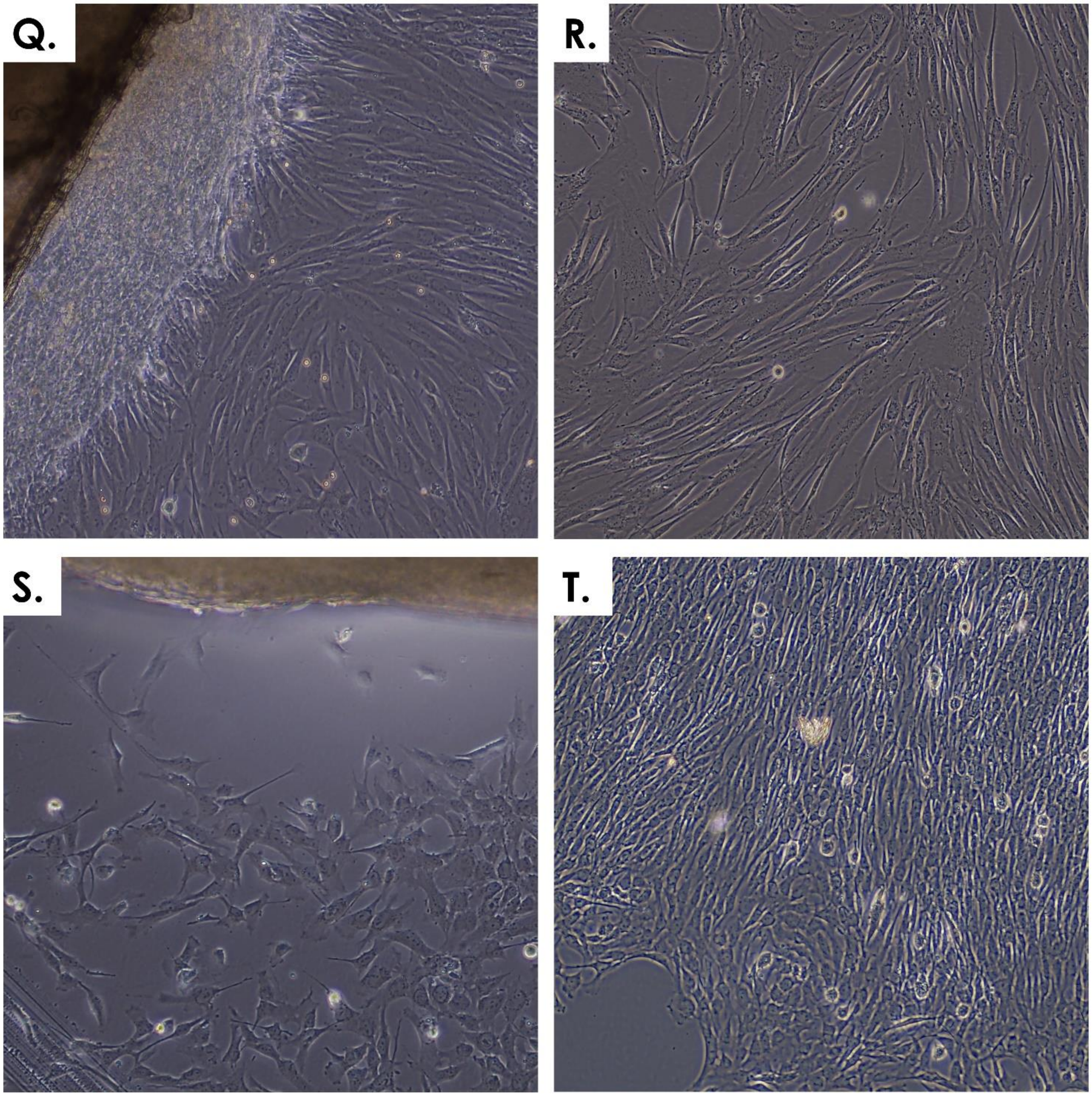

Supplementary Figure S5e | Photographic imaging of FPC *in vitro* culture initiation and culture-expansion steps performed following a single fetal organ donation, within updated legislative frameworks (i.e., post-2007) in Switzerland. Various fetal tissue biopsies were procured from the same organ donation (i.e., donation FE002, 2009) for simultaneous and differential processing, following both enzymatic and non-enzymatic methodologies for *in vitro* cell culture initiation. Pictures were obtained under 200X optical magnification on a phase contrast microscope and represent the non-enzymatically isolated primary FPC types. **(Q-R)** Fetal muscle tissue with emitting muscle FPCs (i.e., FE002-Mu cell type, P0) and expanding cells at P2. **(S-T)** Fetal connective tissue with emitting connective tissue FPCs (i.e., FE002-CT cell type, P0).

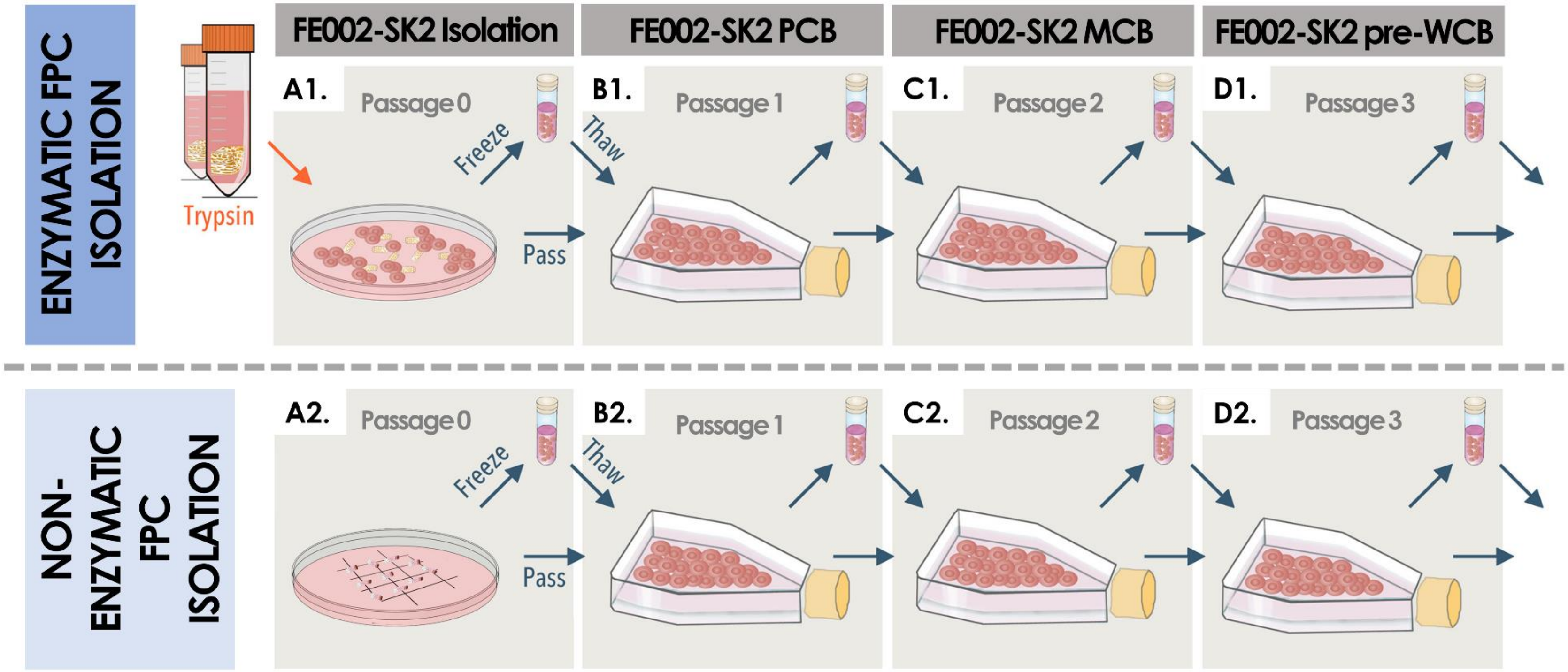

Supplementary Figure S6 | Schematic representation of *in vitro* FPC culture initiation following the enzymatic and non-enzymatic methods and definition of passage number nomenclature within the Swiss Fetal Progenitor Cell Transplantation Program. As nomenclature varies between different groups with regard to passage numbers, a comprehensive definition is provided hereafter. (**A**) "Passage 0" describes cells that have emitted from the fetal tissue fragments or cells that have been plated after enzymatic isolation, which have expanded *in vitro* to confluency. Cells retain the "Passage 0" appellation after having been enzymatically harvested and suspended in medium or buffer in view of seeding in new cell culture vessels. They also retain the same appellation once they have been cryopreserved. (**B**) Therefore, the cells become "Passage 1" when they are directly seeded in new cell culture vessels for further expansion after harvest from the primary tissue culture dishes or when "Passage 0" cryovials are initiated for cell culture vessels seeding. The exact "passage" point is therefore not defined at the point of enzymatic cell detachment, but rather at the time of seeding of a new cell culture surface, which constitutes a better suiting description of the process, as cells are "passed to" a new cell culture environment. (**C-D**) The same method is used to define all subsequent passage numbers. The same logic applies for further incremental modifications of passage numbers along the cycles of serial *in vitro* cellular expansions, possibly interrupted by cryopreservation phases. It is to note that Passage 0 cells are always expanding in tissue culture Petri dishes, in both cell isolation workflows (i.e., enzymatic and non-enzymatic), before passaging to cell culture flasks. Additionally, for consistency and coherence of the passage nomenclature within the multi-tiered FPC banking system, the *in vitro* passages homogenously describe cell expansions and harvest procedures characterized by constant relative viable cell seeding densities, population doubling values, and doubling times. Therefore, within the defined *in vitro* lifespan of the FPC type of interest, cells are always seeded in the same way and should grow to attain confluency in a defined and constant number of days, given that nutritive medium is exchanged at regular and constant intervals (i.e., twice or thrice *per* week). The use of passage numbers rather than population doubling values is coherent with the optimized expansion protocols applied to primary FPC culture, as the number of "gentle" enzymatic treatments (i.e., harvest by trypsinization) is minimized, but the number of population doublings is relatively elevated in comparison to other cell types, as FPCs support relatively low cell seeding densities (e.g., 10^3 - 3 x 10^3 viable cells/cm^2, cell type-dependant) and relatively high endpoint cell yields at harvest (e.g., 10^4 - 10^5 total cells/cm^2, cell type-dependant). **FPC** Fetal Progenitor Cell; **MCB** Master Cell Bank; **PCB** Parental Cell Bank; **WCB** Working Cell Bank.

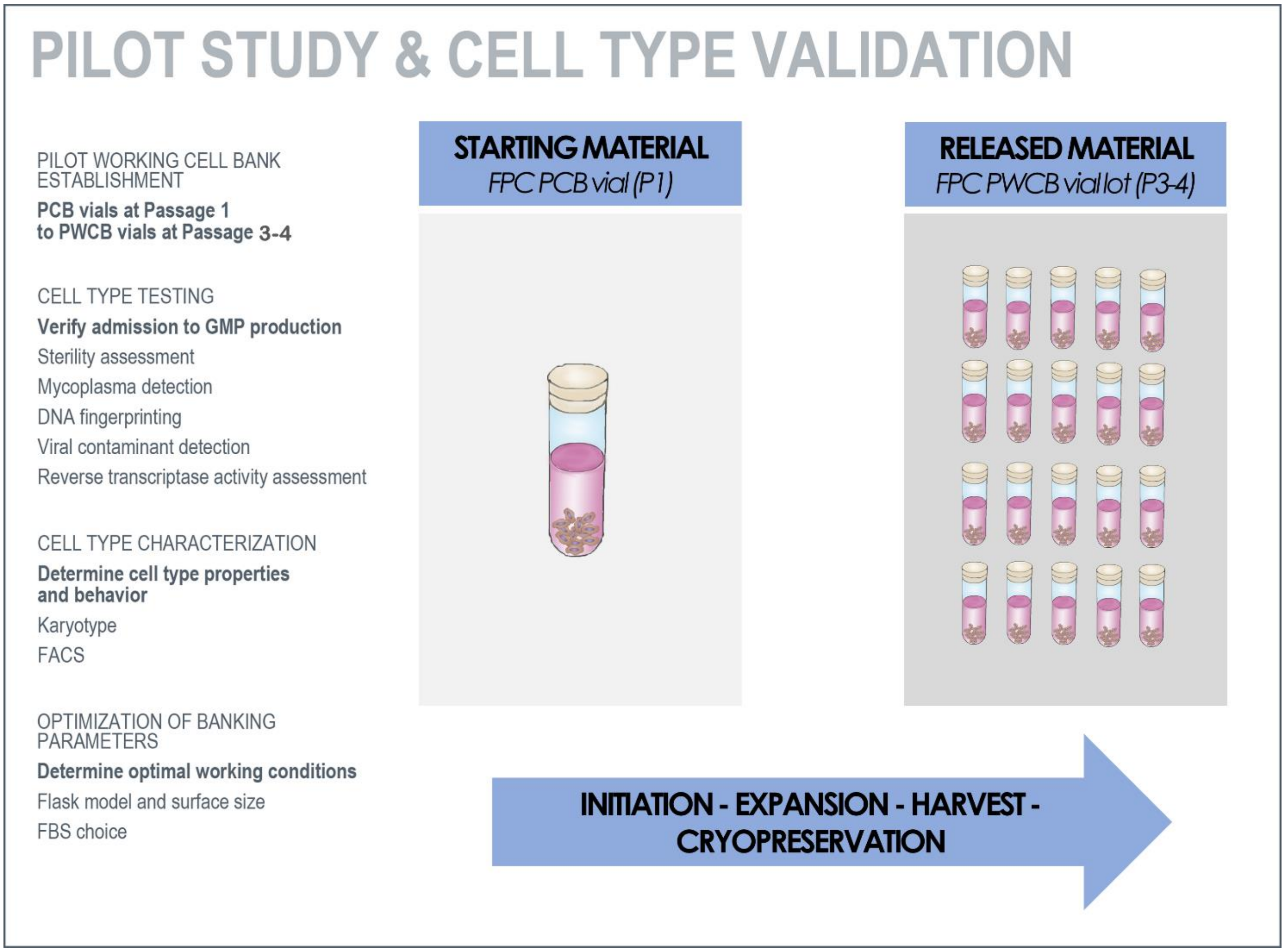

Supplementary Figure S7a | Schematic overview of a pilot study and primary FPC type validation within GMP production workflows, with dermal FPC types provided as an example (e.g., FE002-SK2 cell type). In order to sparingly use the limited stock of PCB vials (P1), part of the Parental Cell Bank is used in a recovery procedure to establish a Pilot Working Cell Bank (PWCB) at Passage 3 or 4. The PWCB is firstly used to determine the admissibility of the considered FPC type to GMP production, through rigorous screening and testing. Secondly, characterization of the FPC type of interest is performed, in order to confirm cell identity, population purity, and cellular characteristics. Thirdly, the PWCB is used for optimization of the cell culture expansion conditions by selecting the optimal growth parameters for the defined FPC type (e.g., type and surface size of culture vessels, FBS brand and lot). **FACS** Fluorescence-Activated Cell Sorting; **FBS** Fetal Bovine Serum; **GMP** Good Manufacturing Practices.

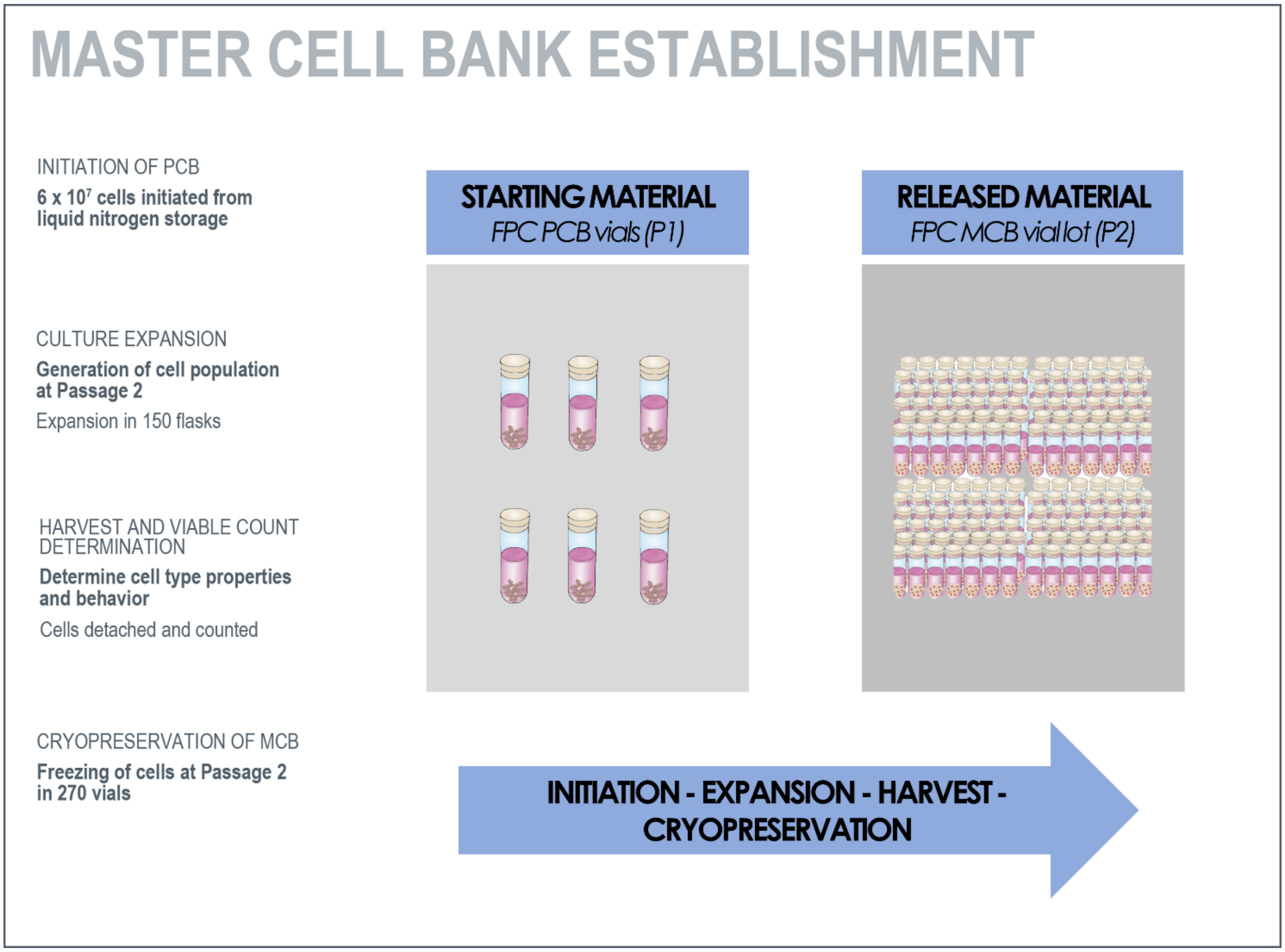

Supplementary Figure S7b | Schematic overview of a Master Cell Bank (MCB) establishment within GMP production workflows, with dermal FPC types provided as an example (e.g., FE002-SK2 cell type). In the example, PCB vials (P1) are initiated and FPCs are culture-expanded once before harvest and cryopreservation. Following appropriate testing, the resulting MCB vial lot may be released. In this example, quantitative data are based on the use of T150 cell culture flasks (i.e., 150 cm^2 culture surfaces) and cryovial contents of 10^6 to 10^7 viable cells at the time of freezing.

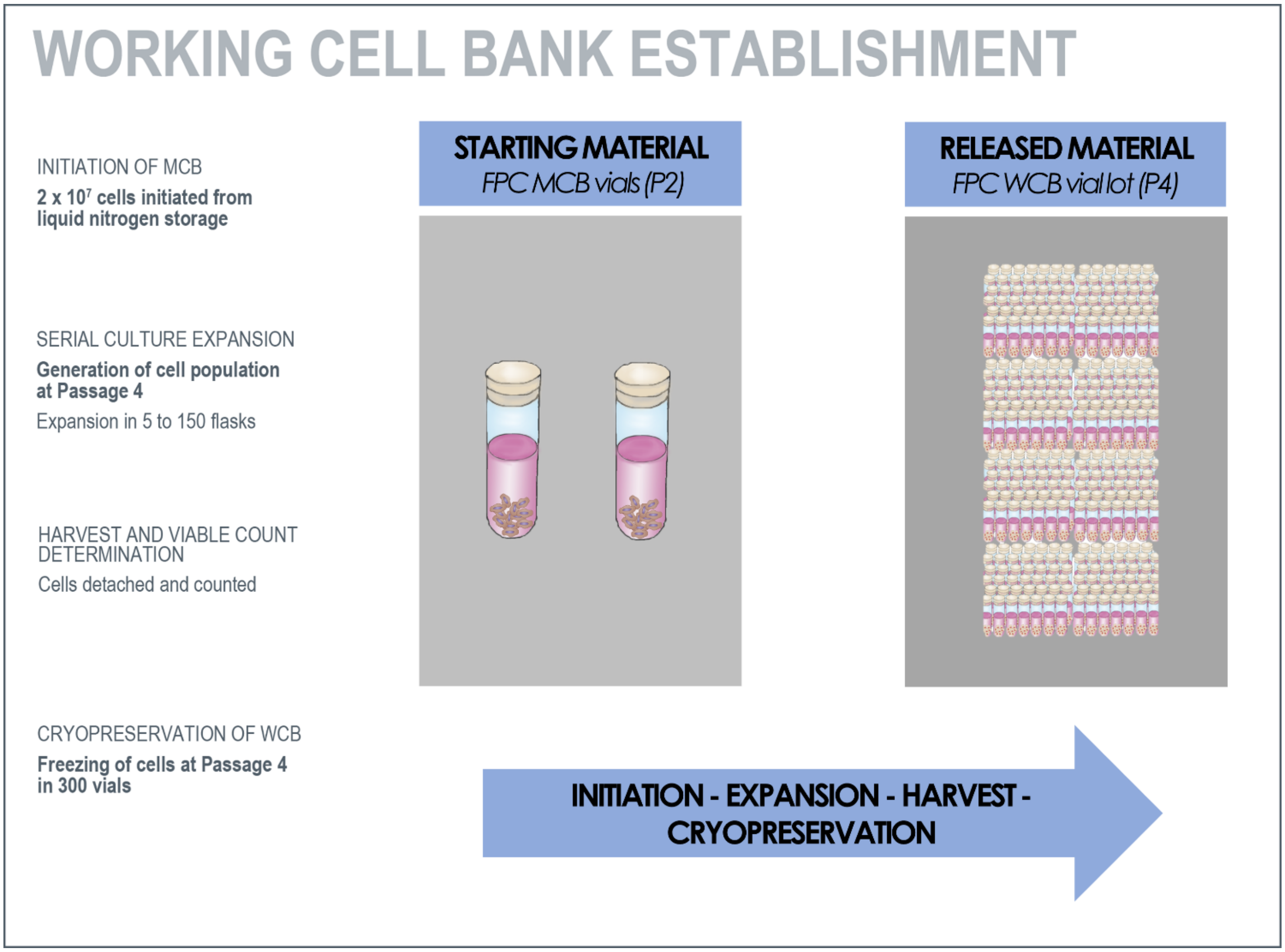

Supplementary Figure S7c | Schematic overview of a Working Cell Bank (WCB) establishment within GMP production workflows, with dermal FPC types provided as an example (e.g., FE002-SK2 cell type). In the example, MCB vials (P2) are initiated and cells are culture-expanded in two phases (i.e., two distinct serial *in vitro* culture expansions) before harvest and cryopreservation. Following appropriate testing, the resulting WCB vial lot may be released. In this example, quantitative data are based on the use of T150 cell culture flasks (i.e., 150 cm^2 culture surfaces) and cryovial contents of 10^6 to 10^7 viable cells at the time of freezing.

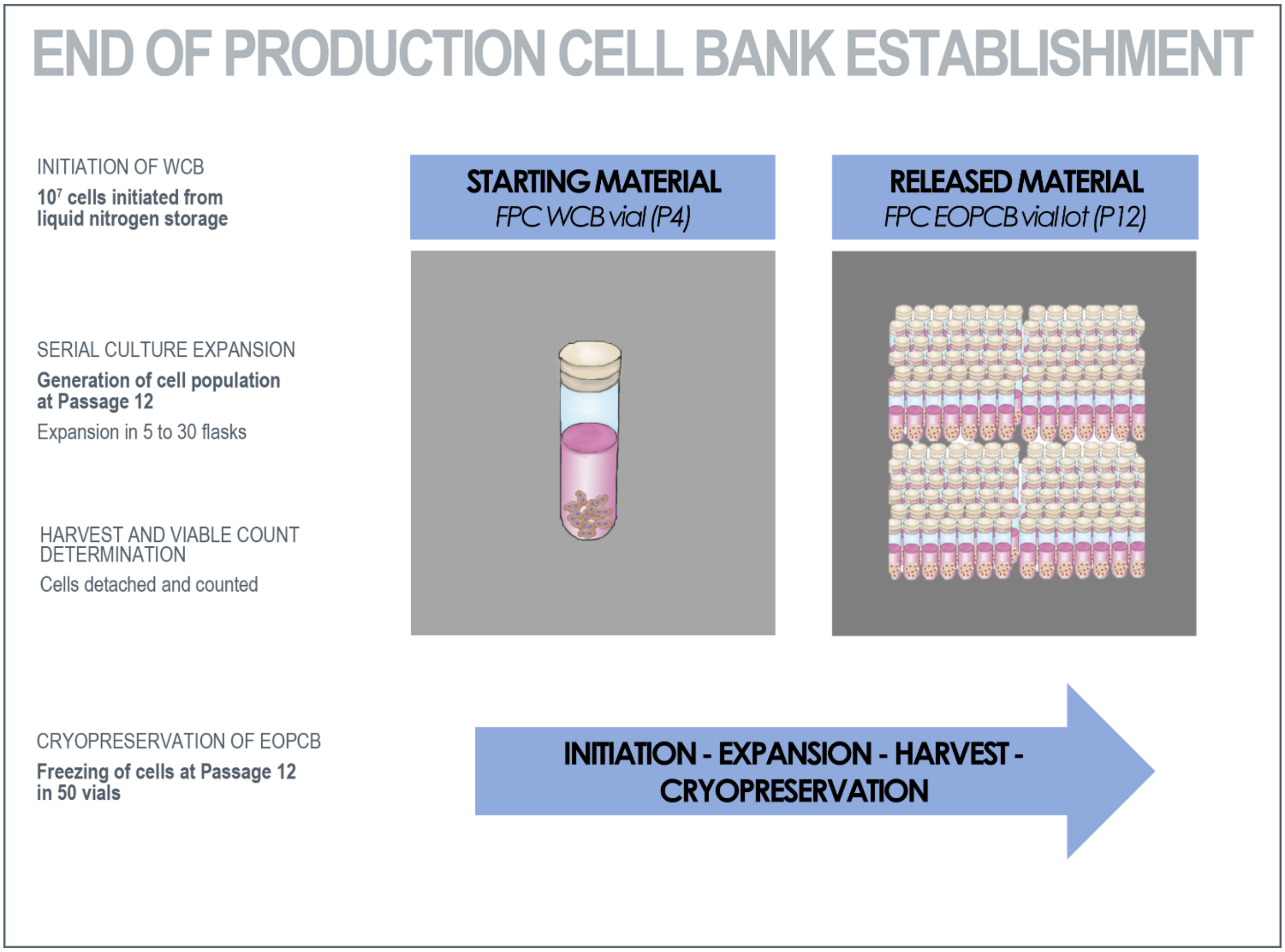

Supplementary Figure S7d | Schematic overview of an End of Production Cell Bank establishment within GMP production workflows, with dermal FPC types provided as an example (e.g., FE002-SK2 cell type). In the example, a WCB vial (P4) is initiated and FPCs are serially culture-expanded (i.e., multiple distinct and successive *in vitro* culture expansions) before harvest and cryopreservation. Following appropriate lot screening and testing, safety of the considered FPC type may be assessed. In this example, quantitative data are based on the use of T150 cell culture flasks (i.e., 150 cm^2 culture surfaces) and cryovial contents of 10^6 to 10^7 viable cells at the time of freezing.

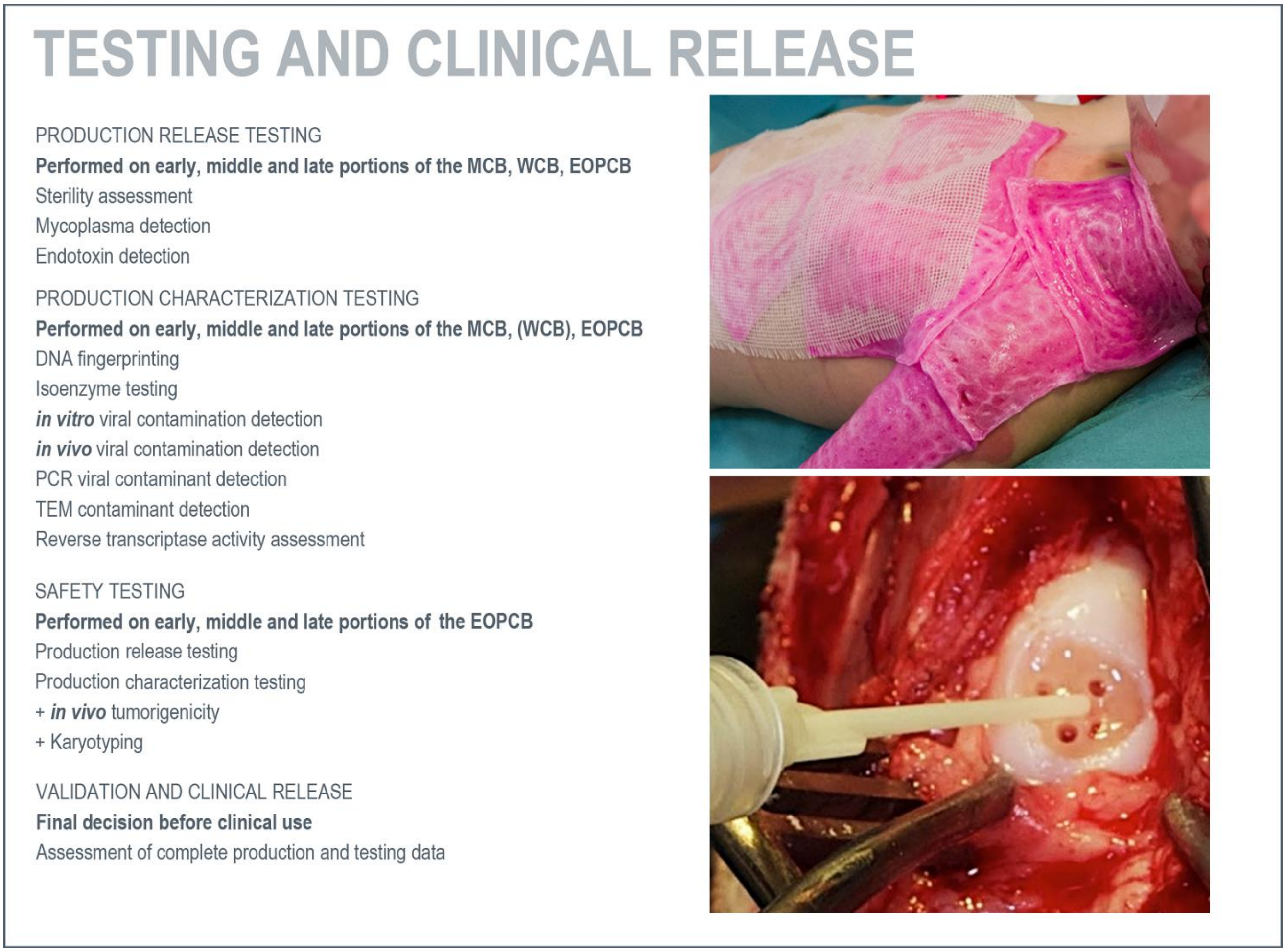

Supplementary Figure S7e | Summary of the different types of testing performed on the various tiers of the FPC production batches within standardized GMP product workflows. The appropriate testing panels are performed when appropriately defined in the multi-tiered production process, guaranteeing the consistency, quality, and safety of the vial lots released by the GMP manufacturing facility. Compilation of production and testing reports allows for review and evaluation of the considered FPC stocks in view of further product development or clinical application (e.g., thermal burn wound care using dermal FPCs or cartilage defect care using cartilage FPCs, top and bottom illustrations, respectively). **DNA** Deoxyribonucleic Acid; **EOPCB** End of Production Cell Bank; **GMP** Good Manufacturing Practices; **MCB** Master Cell Bank; **PCR** Polymerase Chain Reaction; **TEM** Transmission Electron Microscopy; **WCB** Working Cell Bank.
